# Supplementary material for: Glycated haemoglobin A1c (HbA1c) for detection of diabetes mellitus and impaired fasting glucose in Malawi: a diagnostic accuracy study
Source: BMJ Open. 2018 May 5;8(5):e020972. doi: 10.1136/bmjopen-2017-020972 (PMC5942411; doi:10.1136/bmjopen-2017-020972)
Supplement: Supplementary file 1 [file bmjopen-2017-020972supp001.pdf]

```
*******************
*** Hb1Ac AUROC ***
*******************
      use "30Aug17.dta", clear
      keep gluc_units hba1_units ///
            BMI systolic diastolic chol_units tg_units hdl ldl hgb ///
            sex site stid age
      qui compress
      sum

****************
*** NEW VARS ***
****************************************************************
      gen diabetes_fpg=(gluc_units>=7.0)
      label variable diabetes_fpg "Newly diabetes positive (fasting plasma glucose>=7)"
      label define yesno 0 No 1 Yes
      label values diabetes_fpg yesno

      recode gluc_units (min/6.09=0)(6.10/6.99=1)(7.0/max=0), gen(prediabetes)
      label variable prediabetes "Pre-diabetes (fasting glucose of 6.1-6.9)"
      label values prediabetes yesno

      recode gluc_units (min/5.59=0)(5.6/max=1), gen(gluc56)
      label variable gluc56 "Fasting glucose >=5.6 mmol/L"
      label values gluc56 yesno

      recode hba1 (min/6.49=0)(6.50/max=1), gen(hba65)
      label variable hba65 "Diabetes screen positive (HbA1c >= 6.5%)"
      label values hba65 yesno

      recode hba1 (min/6.79=0)(6.80/max=1), gen(hba68)
      label variable hba68 "Diabetes screen pos (HbA1c >=6.8%)"
      label values hba68 yesno

      gen hbaint = round(hba1_units,1)
      label variable hbaint "HbA1c score, rounded to integer"

      gen hbadec = round(hba1_units,0.1)
```

```
label variable hbaint "HbA1c score, rounded to tenths"

encode sex, gen(gender)
label define gender 1 Female 2 Male, modify
label values gender gender
label variable gender "Sex"
drop sex
rename gender sex

gen tempsite = site=="L"
label define site 0 Karonga 1 Lilongwe, modify
label values tempsite site
drop site
rename tempsite site
label variable site "Research site"

gen hbatest = !mi(hba1)
gen pw =!mi(hba1)
recode pw (1=10) if gluc_units<5.6
label variable pw "Probability sampling weight for HbA1c testing"

sort stid
gen pid = _n
label variable pid "Unique identifier"
expand=pw
summarize hba1_unit if diabetes_fpg==0
gen meanhba = r(mean)
gen sdhba = r(sd)
gen zscore = (hba1_units - meanhba) / sdhba
label variable zscore "HbA1c z-score"
notes zscore: Using only HbA1c scores from diabetes-negative pts and re-weighted for the sub-sampling
duplicates drop pid, force

gen zdec = round(zscore, 0.1)
label variable zdec "Normal HbA1c z-score, rounded to 0.1"

recode BMI (min/24.9999=0)(25/29.999=1)(30/max=2), gen(bmi_3c)
label define bmi 0 Normal 1 Overweight 2 Obese
```

```
      label values bmi_3c bmi
      label variable bmi_3c "BMI category"

      gen anemia=0
      replace anemia=1 if sex==2 & hgb<13.5
      replace anemia=1 if sex==1 & hgb<12.0
      label values anemia yesno

      quiet compress

      tempfile full
      save `full'
*** END NEW VARS ***********************************************

*** FLOW CHART ***
      use `full', clear
      count
      drop if mi(gluc_units)
      drop if mi(hba1)
      tab gluc56, sum(hba1)

**************************
*** DESCRIPTIVE CHECKS ***
**************************
      sum age sex site hba1 gluc_units diabetes_fpg pw    /* Most of these are skewed */
      logistic diabetes_fpg hba1_units [pw=pw]
      prop diabetes_fpg
      prop diabetes_fpg [pw=pw]


****************************************
*** TABLE 1: Patient characteristics ***
****************************************
      tabstat age sex BMI syst dias chol_units tg_units hdl_units ldl_units hgb hba1_units gluc_units[aw=pw],
stat(n p50 p25 p75) by(site) f(%9.2f) col(s)
      foreach var of varlist sex bmi_3c anemia hba65 prediabetes diabetes_fpg {
            prop `var' [pw=pw]
            prop `var' [pw=pw], over(site)
```

```
		}
	replace prediabetes=. if gluc_units>=7.0

*******************************
*** TABLE 2: Validity stats ***
*******************************
	* Overall dataset for HbA1c vs diabetes
	foreach x of varlist diabetes_fpg prediabetes {
		somersd `x' hba1 [pw=pw], trans(c)
		roctab `x' hba1, binomial sum
		senspec `x' hbadec [pw=pw], sensitivity(sens) specificity(spec) ntpos(tp) nfpos(fp) ntneg(tn) 
nfneg(fn)
		for var sens spec tp fp tn fn: replace X = 100*X
		gen ppv = 100*tp / (tp+fp)
		gen npv = 100*tn / (tn+fn)
		gen lrpos = sens / (100-spec)
		gen lrneg = (100-sens) / spec
		gen jtemp = sens + spec - 100
		egen youdenj = max(jtemp)
		replace youdenj=. if youdenj!=jtemp
		table zdec, c(mean sens mean spec mean youdenj mean lrpos mean lrneg) format(%9.2f)
		drop sens spec tp fp tn fn ppv npv lrpos lrneg jtemp youdenj
	}

***************
*** TABLE 3 ***
***************
	* Stratum-specific HbA1c vs diabetes
	foreach var of varlist site sex bmi_3c anemia {
		levelsof `var', local(levels)
		foreach l of local levels {
			tab `var' if `var'==`l'
			somersd diabetes_fpg hba1 [pw=pw] if `var'==`l', trans(c)
			senspec diabetes_fpg hbadec [pw=pw] if `var'==`l', sensitivity(sens) specificity(spec)
			for var sens spec: replace X = 100*X
			gen lrpos = sens / (100-spec)
			gen lrneg = (100-sens) / spec
```

```
                  table zdec if zdec>1.66 & zdec<1.71 & `var'==`l', c(mean sens mean spec mean lrpos mean
lrneg) format(%9.2f)
                  drop sens spec lrpos lrneg
                  }
            }

*******************************
*** Table 4: Diabetes chars ***
*******************************
* Present clinical charteristics of diabetes+ by fasting glucose criteria
      tabstat age sex BMI syst dias chol_units tg_units hdl_units ldl_units hgb hba1_units gluc_units  if
diabetes_fpg [aw=pw], stat(p50 p25 p75) f(%9.2f) col(s)
      prop sex [pw=pw] if diabetes_fpg

* Present clinical characteristics among HbA1c+, by true status
      tab hba65 diabetes_fpg
      tabstat age sex BMI syst dias chol_units tg_units hdl_units ldl_units hgb hba1_units gluc_units  if hba65
[aw=pw], by(diabetes_fpg) stat(p50 p25 p75) f(%9.2f) col(s)
      prop sex if hba65 [pw=pw], over(diabetes_fpg)
```
