# Supplementary material for: Glycated haemoglobin A1c (HbA1c) for detection of diabetes mellitus and impaired fasting glucose in Malawi: a diagnostic accuracy study
Source: BMJ Open. 2018 May 5;8(5):e020972. doi: 10.1136/bmjopen-2017-020972 (PMC5942411; doi:10.1136/bmjopen-2017-020972)
Supplement: Author's manuscript [file bmjopen-2017-020972.draft_revisions.pdf]

BMJ Open is committed to open peer review. As part of this commitment we make the peer review history of every article we publish publicly available.

When an article is published we post the peer reviewers' comments and the authors' responses online. We also post the versions of the paper that were used during peer review. These are the versions that the peer review comments apply to.

The versions of the paper that follow are the versions that were submitted during the peer review process. They are not the versions of record or the final published versions. They should not be cited or distributed as the published version of this manuscript.

BMJ Open is an open access journal and the full, final, typeset and author-corrected version of record of the manuscript is available on our site with no access controls, subscription charges or pay-per-view fees (http://bmjopen.bmj.com).

If you have any questions on BMJ Open's open peer review process please email info.bmjopen@bmj.com

BMJ Open

Glycated hemoglobin A1c (HbA1c) for detection of pre-diabetes and diabetes mellitus in Malawi: a diagnostic accuracy study

Journal:	*BMJ Open*
Manuscript ID	bmjopen-2017-020972
Article Type:	Research
Date Submitted by the Author:	05-Dec-2017
Complete List of Authors:	Rathod, Sujit; London School of Hygiene and Tropical Medicine, Department of Population Health<br>Crampin, Amelia; London School of Hygiene and Tropical Medicine<br>Musicha, Crispin; Malawi Epidemiology and Intervention Research Unit<br>Kayuni, Ndoliwe; Malawi Epidemiology and Intervention Research Unit<br>Banda, Louis; Malawi Epidemiology and Intervention Research Unit<br>Saul, Jacqueline; London School of Hygiene and Tropical Medicine<br>McLean, Estelle; London School of Hygiene and Tropical Medicine; Malawi Epidemiology and Intervention Research Unit<br>Branson, Keith; London School of Hygiene and Tropical Medicine<br>Jaffar, Shabbar; Liverpool School of Tropical Medicine<br>Nyirenda, Moffat; London School of Hygiene and Tropical Medicine
Keywords:	HbA1c, Type 2 diabetes, Malawi, validity, receiver operating characteristic

SCHOLARONE™
Manuscripts

Glycated hemoglobin A_{1c} (HbA_{1c}) for detection of pre-diabetes and diabetes mellitus in Malawi: a diagnostic accuracy study

Sujit D Rathod*,[1] Amelia C Crampin,[1] Crispin Musicha,[2] Ndoliwe Kayuni,[2] Louis Banda,[2] Jacqueline Saul,[1] Estelle McLean,[1,2] Keith Branson,[1] Shabbar Jaffar,[3] Moffat J Nyirenda,[1]

[1] London School of Hygiene & Tropical Medicine, London, United Kingdom

[2] Malawi Epidemiology and Intervention Research Unit, Chilumba, Malawi

[3] Liverpool School of Tropical Medicine, Liverpool, United Kingdom

*Corresponding author
Department of Population Health
London School of Hygiene & Tropical Medicine
Keppel Street
London WC1E7HT
United Kingdom
P: +44 020 7299 4683
F: +44 (0)20 7436 4230
sujit.rathod@lshtm.ac.uk

For peer review only - http://bmjopen.bmj.com/site/about/guidelines.xhtml

Abstract

Objectives: A key research question for type 2 diabetes control in sub-Saharan Africa concerns improving the accessibility of diagnostics. HbA_{1c} testing has logistic advantages over fasting plasma glucose and oral glucose tolerance tests but its utility in African populations is unclear.

Design: We conducted a validation study of HbA_{1c} to detect type 2 diabetes, using fasting plasma glucose (FPG) >=7.0 mmol/L as the reference standard.

Participants: 3645 adults (of whom 63% female) recruited from sites in urban and rural Malawi.

Results: HbA_{1c} demonstrated excellent validity to detect FPG-defined diabetes, with an area under the receiver operating characteristic (AUROC) curve of 0.92 (95% CI 0.90-0.94). At HbA_{1c}≥6.5% (48 mmol/mol), sensitivity was 78.7% and specificity was 94.0%. Subgroup AUROCs ranged from 0.86 for participants with anemia to 0.94 for participants in urban Malawi. There were clinical and metabolic differences between participants who were true diabetes vs. false positives when HbA_{1c}≥6.5% (48 mmol/mol).

Conclusions: The findings from this study provide justification to use HbA_{1c} to detect type 2 diabetes. As HbA_{1c} testing is substantially less burdensome to patients than either fasting plasma glucose testing or oral glucose tolerance testing, it represents a useful option for expanding access to diabetes care in sub-Saharan Africa.

For peer review only - http://bmjopen.bmj.com/site/about/guidelines.xhtml

Strengths and limitations of this study

- Only two studies have assessed the validity of HbA_{1c} to detect type 2 diabetes in African-origin populations; this is the first such study from Malawi.
- We used fasting plasma glucose as the reference standard, which is commonly used in clinical practice and for validation studies.
- The results of this study may not be generalizable to other populations in sub-Saharan Africa, or those with anemia.

For peer review only

For peer review only - http://bmjopen.bmj.com/site/about/guidelines.xhtml

Over 400 million people live with type 2 diabetes worldwide, with more than 75% of these in low- and middle-income countries (1). While the prevalence of diabetes in sub-Saharan African is lower than in other global regions, around 6-9% (2,3), demographic models project that this region will experience the fastest growth rate in cases over the next 20 years (1). Approximately 50-66% of people with diabetes are undiagnosed (1,4), and complications are common even after diagnosis (5). As barriers to diabetes care become surmounted in this region (4), a key research question for diabetes control concerns improving the accessibility of diagnostics.

The diagnosis of diabetes has traditionally been based upon the detection of elevated plasma glucose levels, either after fasting or two hours after an oral glucose tolerance test, or, in symptomatic individuals, after a random blood glucose check (6). While these tests are available in sub-Saharan Africa, access to services are limited, and travel times and clinic waiting times are high, integrity of the samples and quality of the measuring tools is in most case uncertain. More reliable approaches to circumvent some of these challenges are required. Recently, the American Diabetes Association (ADA) and the World Health Organization (WHO) have recommended using glycated hemoglobin A_{1c} (HbA_{1c}) in blood to diagnose diabetes mellitus (6,7). HbA_{1c} testing provides significant practical advantages over glucose testing as it does not fluctuate appreciably and thus can be performed at any time of the day. Further, HbA_{1c} testing does not require any special pre-test preparations, such as overnight fasting or glucose loading.

There are acknowledged limitations when attempting to measure HbA_{1c} levels which may be relevant for diagnostics in sub-Saharan Africa (6,8). It is unclear whether one can use HbA_{1c} to diagnose diabetes for people who have malaria (6), haemolytic anaemia (9,10),

For peer review only - http://bmjopen.bmj.com/site/about/guidelines.xhtml

sickle cell anaemia (11), HIV infection (12,13) or who are of African descent (14,15). Given uncertainty around the validity of HbA_{1c} to diagnose type 2 diabetes and pre-diabetes in sub-Saharan Africa, we conducted a diagnostic accuracy study in Malawi, and have reported results here.

Design and Methods

Study setting

This report uses data collected as part of a Malawi Epidemiology and Intervention Research Unit (MEIRU) survey, which aimed to understand the burden and risk factors of non-communicable diseases in Malawi (16), where the national prevalence of diabetes is estimated to be 5.6% (17). Community-based cross-sectional surveys were conducted in Karonga District (May 2013-April 2016) (18) and Lilongwe City (June 2013-April 2017) (16). Karonga is a rural, low-altitude, malaria-endemic district in northern Malawi, and Lilongwe is an urban, high altitude city in central Malawi with low malaria prevalence.

Recruitment and data collection

Detailed study procedures have been previously reported (16). All adults aged 18 years and above who were usually resident in either of the study sites were eligible to participate in the parent study. All households were approached consecutively and all residents aged ≥18 years were recruited. Adults could provide informed consent for any or all parts of the study visit, which was comprised of a standardized interview, physical measurements, and laboratory specimen collection (including specimens for FPG and

For peer review only - http://bmjopen.bmj.com/site/about/guidelines.xhtml

For peer review only

HbA_{1c} testing). Quantitative determination of fasting plasma glucose (hexokinase method) and and HbA_{1c} was performed using the Beckman Coulter Chemistry Analyzer AU480 system according to the manufacturer's guidelines.

FPG testing was conducted on all participant samples. HbA_{1c} testing was conducted on all participants samples for which FPG results were greater than 5.6 mmol/L and in a 10% random sample of those with FPG lower than 5.6 mmol/L. The lab technician who conducted the HbA_{1c} test was blind to the FPG result. Nearly all (87%) of HbA_{1c} tests were completed within 24 hours of FPG test. The cut-points recommended by the ADA to define fasting plasma glucose (FPG) as normal (<6.1 mmol/L), impaired/pre-diabetes (6.1 to 6.9 mmol/L) or diabetes (≥7.0 mmol/L) (19) were used. Categories of body mass index (BMI) for underweight/normal (BMI<25kg/m^2), overweight (25≤BMI<30) and obese (BMI≥30), and categories for anemia (haemoglobin<12.0g/dL for women and <13.5 for men) were used.

Statistical analyses

Participants who reported a past diagnosis of diabetes or history of taking diabetes medication were excluded from this analysis. First, demographic and clinical variables (i.e. age, sex, location, BMI, blood pressure, plasma glucose and lipid profile, and HIV serostatus) were described separately for the Karonga and Lilongwe samples. Medians and interquartile ranges were reported for the continuous measures, most of which were skewed, and proportions for categorical measures. Second, distribution of HbA_{1c} level was compared by FPG result (<7.0 vs >7.0 mmol/L) with logistic regression. Third, the validity of HbA_{1c} for diagnosing diabetes, using FPG (>7.0 mmol/L) as reference standard, was assessed by using Somers' D statistic with Harrell's C transformation to

For peer review only - http://bmjopen.bmj.com/site/about/guidelines.xhtml

estimate the area under the receiver operating characteristics (AUROC) curve and 95% confidence interval. Validity statistics (i.e. sensitivity, specificity, and positive and negative likelihood ratios) were reported for standardized Hb1Ac scores which correspond to 1.0, 2.0, 3.0 and 4.0 standard deviations above the sample's mean HbA_{1c} score, at an 'optimal' value identified by the Youden's J statistic (where the sum of the sensitivity and specificity reaches its maximum) (20), and at 6.5% (48 mmol/mol), which is the value recommended by several diabetes associations. The standardized values were generated using the distribution of HbA_{1c} scores from the diabetes-negative participants with HbA_{1c} test results (i.e. all participants with fasting plasma glucose between 5.6-7.0 mmol/L and a 10% sub-sample of participants with plasma glucose <5.6) such that the sub-sampled participants were upweighted 10-fold before generating the distribution. Fourth, to consider the validity of HbA_{1c} to detect diabetes across subgroups, the ROC analysis was stratified by site, sex, BMI, and hemoglobin level. Within each stratum the sensitivity, specificity, positive and negative likelihood ratio when using HbA_{1c}≥6.5% (48 mmol/mol) as a cut off score were reported. Fifth, the AUROC and 95% CI for detecting pre-diabetes (FPG ≥ 5.6 mmol/L) with HbA_{1c} among those who did not have diabetes (FPG <7.0 mmol/L) was estimated. Finally, the clinical characteristics were described for the subsets of participants who had FPG-defined diabetes, and for true- and false-positive participants with HbA_{1c}≥6.5% (48 mmol/mol). Again, medians and interquartile ranges were reported for continuous measures and proportions for categorical measures. The analysis was conducted using Stata SE 14.2 (StataCorp, College Station, Texas, USA)(See Stata code in supplemental material) with complete-

For peer review only - http://bmjopen.bmj.com/site/about/guidelines.xhtml

case analysis. Aside count figures, the statistical results were weighted by the inverse probability of receiving the HbA_{1c} test.

Ethical approval

The study protocol was reviewed and approved by the Malawi National Health Sciences Research Committee, and London School of Hygiene and Tropical Medicine (UK) Ethics Committee. Participants identified as having diabetes were referred to chronic care clinics established in conjunction with the Malawi Ministry of Health, where they initiated management per national treatment guidelines.

Results

As shown in the STARD flow diagram (Figure 1), 30,574 adults consented to the interview and clinical examination in the parent study, of whom 27,846 also provided consent for blood specimen collection, as was required for this sub-study. No participant experienced a severe adverse event due to the specimen collection. This analysis includes the 3645 participants (1613 in Karonga and 2032 in Lilongwe) who had both FPG and HbA_{1c} levels measured, and who did not report prior diagnosis of diabetes by a medical professional.

Demographic and Clinical Characteristics

The demographic and clinical characteristics of analysis participants are reported in Table 1.

For peer review only - http://bmjopen.bmj.com/site/about/guidelines.xhtml

For the 3645 participants, the median age was 33 years (IQR 24-44) and 63% were female. The median HbA_{1c} level was 5.3% (IQR 4.9-5.7) and 7.3% had HbA_{1c} level of 6.5% (48 mmol/mol) or higher. The median FPG level was 4.7 mmol/L (IQR 4.3-5.0); 1.8% of participants had a FPG level consistent with pre-diabetes and another 1.7% with diabetes.

Diagnostic validity of HbA_{1c}

For every percentage unit increase in HbA_{1c} score, the odds of having FPG-defined diabetes increase by 2.80 times (95% CI 2.21-3.53). Further HbA_{1c} demonstrated excellent validity to detect for FPG-defined diabetes, with an AUROC of 0.92 (95% CI 0.90-0.94). Validity statistics are presented for a range of HbA_{1c} cut points in Table 2.

An HbA_{1c} value of 6.0% corresponded to 1.0 standard deviation above the mean in the diabetes-negative sample's distribution of HbA_{1c} scores; at this cut point 84.4% of people with diabetes would test positive (sensitivity) and 86.9% of people without diabetes would test negative (specificity). At the commonly-recommended HbA_{1c} threshold of 6.5% (48 mmol/mol), sensitivity was 78.7% and specificity was 94.0%. Youden's J was at an HbA_{1c} value of 6.6%, where sensitivity was 78.3% and specificity was 94.6%. Stratum-specific AUROCs (Table 3), ranged from a minimum of 0.86 for participants with anemia to a maximum of 0.94 for participants in Lilongwe. With $HbA_{1c} \geq 6.5\%$ (48 mmol/mol), participants with anemia had 61.5% sensitivity, 96.4% specificity and a positive likelihood ratio of 17.4, and the corresponding figures for participants in Lilongwe were 70.2%, 95.4%, and 15.2.

For peer review only - http://bmjopen.bmj.com/site/about/guidelines.xhtml

Among participants who did not have FPG-defined diabetes, the HbA_{1c} score had average validity to detect pre-diabetes (AUROC 0.70, 95% CI 0.67-0.73).

Clinical characteristics of participants meeting diagnostic criteria for diabetes

The clinical characteristics of participants who had FPG-defined diabetes, and for participants who had a true positive and false positives results are presented in Table 4.

The participants who had FPG-defined diabetes (FPG ≥7.0 mmol/L) had a median age of 50 years (IQR 41-60) and 65.5% were female. The median BMI was 27.9 kg/m^2 (IQR 24.2-32.5) and the median systolic blood pressure 133.5 mm Hg (IQR 121.5-153). The true positive participants (HbA_{1c} ≥6.5% (48 mmol/mol) and FPG≥7.0 mmol/L) had a median age of 51 years (IQR 43-61) and 66.3% were female. The median BMI was 28.2 kg/m^2 (IQR 24.8-32.5) and the median systolic blood pressure was 135 mm Hg (IQR 123-156). For the false positive participants (HbA_{1c} ≥6.5% (48 mmol/mol) and FPG< 7.0 mmol/L) had a median age of 35 years (IQR 23-51) and 51.0% were female. The median BMI was 24.8 kg/m^2 (IQR 20.9-28.3) and the median systolic blood pressure was 125 mm Hg (IQR 115-136).

Discussion

In a large, multi-site sample of adults in Malawi, we found that HbA_{1c} is highly predictive of FPG-defined type 2 diabetes, a relationship which was consistent across several sub-groups. HbA_{1c} was less predictive for pre-diabetes. Using a cut off value of HbA_{1c} 6.5% (48 mmol/mol) to detect diabetes, there were demographic and clinical differences between true- and false-positive participants.

For peer review only - http://bmjopen.bmj.com/site/about/guidelines.xhtml

For peer review only

This analysis is one of the first to compare HbA_{1c} as a stand-alone test to detect type 2 diabetes in a black African population in sub-Saharan Africa, and the first from Malawi. In Uganda, Mayega et al found that HbA_{1c} had moderate validity (AUROC 0.75) for detecting FPG-defined diabetes (21). These findings from sub-Saharan Africa complement meta-analyses of studies in East Asia, Middle East and Europe which show that HbA_{1c} is a valid test for detecting diabetes (22–24) across different ethnic groups.

There were several notable demographic and clinical differences between participants who were true vs false positives for FPG-defined diabetes at HbA_{1c}≥6.5% (48 mmol/mol). Compared to participants who had HbA_{1c}≥6.5% (48 mmol/mol) and FPG<7.0 mmol/L, those who had both FPG≥7.0 and HbA_{1c}≥6.5% (48 mmol/mol) were more likely to be female, to be older, and to have higher BMI and blood pressure. These findings are consistent with data from the Uganda where the AUROC for all participants was 0.75, but was significantly higher at 0.90 for overweight participants and 0.98 for obese participants. This suggests that HbA1c is particularly useful in in detecting diabetes in individuals with insulin resistance or the metabolic syndrome (constellation of high BMI, hypertension, dyslipidemia). In contrast, the false positive group (HbA_{1c}≥6.5% (48 mmol/mol) and FPG<7.0 mmol/L) lacked features of the metabolic syndrome. This is likely to include individuals with isolated postprandial hyperglycemia, rather than fasting hyperglycemia, (perhaps from pancreatic deficiency rather than insulin resistance) which would require oral glucose tolerance testing (OGTT) to demonstrate. Indeed, HbA_{1c} has also been shown to strongly predict OGTT-defined diabetes in South Africa, where Hird et al estimated the AUROC at 0.94 (25).

For peer review only - http://bmjopen.bmj.com/site/about/guidelines.xhtml

We found that HbA_{1c} had poor predictive ability for detecting pre-diabetes. This is consistent with other studies in Africa and evidence from a recent meta-analysis by Kodama et al (21,24,25). Accordingly, evidence strongly suggests that HbA_{1c} is not appropriate for detecting pre-diabetes.

Stratum-specific AUROC analysis indicated largely consistent values across strata (i.e. by sex, site, and BMI), which is evidence of suitability of HbA_{1c} to detect diabetes across a range of groups. However, AUROC value were lowest for those with anemia – which appears to be driven by a drop in sensitivity. These findings complement those by Kim et al and Son et al (9,10) which call into question the accuracy of HbA_{1c} measures in the presence of anemia. Future research can establish whether anemia contraindicates HbA_{1c} testing, or if a different cut off score is appropriate for people with anemia.

We measured FPG rather than OGTT in the full sample of participants and used it as the reference standard for diabetes in this analysis. FPG is more commonly used in practice as well as in validation studies (21,26), and, moreover, FPG is highly correlated to present and to future diabetes (24). In contrast the frequency of OGTT-derived isolated postprandial hyperglycemia or its clinical course is not clear, particularly in sub-Saharan Africa, and will require further research.

Demographic models indicate that sub-Sahara will experience a substantial increase in diabetes prevalence in the coming years, which will require urgent strategies to scale up detection and treatment in order to increase access to care. As HbA_{1c} testing is less burdensome to patients than FPG and OGTT, it represents a useful option for expanding access to diabetes care. This will become particularly important as countries in sub-

For peer review only - http://bmjopen.bmj.com/site/about/guidelines.xhtml

For peer review only

Saharan Africa allocate increasing resources to the health sector, and as operating costs for the HbA_{1c} test reduce over the next few years.

Funding

The work is supported primarily by the Wellcome Trust, United Kingdom [grant 098610Z/12/Z].

Competing interests

None declared.

Data Sharing

Study data are available on request from the Principle Investigators, Moffat Nyirenda (moffat.nyirenda@lshtm.ac.uk) and Amelia Crampin (mia.crampin@lshtm.ac.uk); the participant consent allows for data sharing with research collaborators. The statistical analysis code is available here as supplemental material.

For peer review only

Author contributions

MJN, SJ and ACC conceived the study. SJ, ACC, CM, NK designed the protocols and implemented the study. LB designed the laboratory protocols and laboratory quality control, and implemented the laboratory work. JS and KB designed the data management protocols. EM designed the data cleaning protocols. SDR conducted data analysis. All authors wrote the manuscript.

Acknowledgments

We are grateful to the Karonga and Lilongwe communities; participants and traditional authorities, for their engagement in this work. This study would also not have been possible without the encouragement and support of the Malawi Ministry of Health, particularly the Karonga and Lilongwe District Health Offices.

For peer review only

For peer review only - http://bmjopen.bmj.com/site/about/guidelines.xhtml

1 STARD checklist

Section & Topic	No	Item	Reported on page #
**TITLE OR ABSTRACT**			
	**1**	Identification as a study of diagnostic accuracy using at least one measure of accuracy (such as sensitivity, specificity, predictive values, or AUC)	1
**ABSTRACT**			
	**2**	Structured summary of study design, methods, results, and conclusions (for specific guidance, see STARD for Abstracts)	2
**INTRODUCTION**			
	**3**	Scientific and clinical background, including the intended use and clinical role of the index test	4
	**4**	Study objectives and hypotheses	5
**METHODS**			
Study design	**5**	Whether data collection was planned before the index test and reference standard were performed (prospective study) or after (retrospective study)	5
Participants	**6**	Eligibility criteria	5
	**7**	On what basis potentially eligible participants were identified (such as symptoms, results from previous tests, inclusion in registry)	5
	**8**	Where and when potentially eligible participants were identified (setting, location and dates)	5
	**9**	Whether participants formed a consecutive, random or convenience series	5
Test methods	**10a**	Index test, in sufficient detail to allow replication	5-6
	**10b**	Reference standard, in sufficient detail to allow replication	5-6
	**11**	Rationale for choosing the reference standard (if alternatives exist)	
	**12a**	Definition of and rationale for test positivity cut-offs or result categories of the index test, distinguishing pre-specified from exploratory	6-7
	**12b**	Definition of and rationale for test positivity cut-offs or result categories of the reference standard, distinguishing pre-specified from exploratory	6
	**13a**	Whether clinical information and reference standard results were available to the performers/readers of the index test	6
	**13b**	Whether clinical information and index test results were available to the assessors of the reference standard	6
Analysis	**14**	Methods for estimating or comparing measures of diagnostic accuracy	6
	**15**	How indeterminate index test or reference standard results were handled	7
	**16**	How missing data on the index test and reference standard were handled	7
	**17**	Any analyses of variability in diagnostic accuracy, distinguishing pre-specified from exploratory	NA
	**18**	Intended sample size and how it was determined	8
**RESULTS**			
Participants	**19**	Flow of participants, using a diagram	Figure 1
	**20**	Baseline demographic and clinical characteristics of participants	Table 1
	**21a**	Distribution of severity of disease in those with the target condition	Table 4
	**21b**	Distribution of alternative diagnoses in those without the target condition	NA
	**22**	Time interval and any clinical interventions between index test and reference standard	6

For peer review only - http://bmjopen.bmj.com/site/about/guidelines.xhtml

Test results	**23**	Cross tabulation of the index test results (or their distribution) by the results of the reference standard	9
	**24**	Estimates of diagnostic accuracy and their precision (such as 95% confidence intervals)	9
	**25**	Any adverse events from performing the index test or the reference standard	8
**DISCUSSION**			
	**26**	Study limitations, including sources of potential bias, statistical uncertainty, and generalisability	12
	**27**	Implications for practice, including the intended use and clinical role of the index test	12
**OTHER INFORMATION**			
	**28**	Registration number and name of registry	NA
	**29**	Where the full study protocol can be accessed	NA
	**30**	Sources of funding and other support; role of funders	12

1

For peer review only

References

1. Ogurtsova K, da Rocha Fernandes JD, Huang Y, Linnenkamp U, Guariguata L, Cho NH, et al. IDF Diabetes Atlas: Global estimates for the prevalence of diabetes for 2015 and 2040. Diabetes Res Clin Pract. 2017 Jun;128:40–50.

2. Hilawe EH, Yatsuya H, Kawaguchi L, Aoyama A. Differences by sex in the prevalence of diabetes mellitus, impaired fasting glycaemia and impaired glucose tolerance in sub-Saharan Africa: a systematic review and meta-analysis. Bull World Health Organ. 2013 Sep 1;91(9):671–682D.

3. NCD Risk Factor Collaboration - Africa Working Group. Trends in obesity and diabetes across Africa from 1980 to 2014: an analysis of pooled population-based studies. Int J Epidemiol [Internet]. 2017 [cited 2017 Jul 19]; Available from: http://fdslive.oup.com/www.oup.com/pdf/production_in_progress.pdf

4. Mbanya JCN, Motala AA, Sobngwi E, Assah FK, Enoru ST. Diabetes in sub-Saharan Africa. The Lancet. 2010 Jun;375(9733):2254–66.

5. Burgess PI, Allain TJ, García-Fiñana M, Beare N a. V, Msukwa G, Harding SP. High prevalence in Malawi of sight-threatening retinopathy and visual impairment caused by diabetes: identification of population-specific targets for intervention. Diabet Med J Br Diabet Assoc. 2014 Dec;31(12):1643–50.

6. The International Expert Committee. International Expert Committee Report on the Role of the A1C Assay in the Diagnosis of Diabetes. Diabetes Care. 2009 Jul 1;32(7):1327–34.

7. American Diabetes Association. Diagnosis and Classification of Diabetes Mellitus. Diabetes Care. 2014 Jan 1;37(Supplement_1):S81–90.

8. Nakanga WP, Crampin A, Nyirenda M. Should haemoglobin A1C be used for diagnosis of diabetes mellitus in Malawi? Malawi Med J. 2016 Apr 6;28(1):28.

9. Son JI, Rhee SY, Woo J, Hwang JK, Chin SO, Chon S, et al. Hemoglobin A1c May Be an Inadequate Diagnostic Tool for Diabetes Mellitus in Anemic Subjects. Diabetes Metab J. 2013;37(5):343.

10. Kim C, Bullard KM, Herman WH, Beckles GL. Association Between Iron Deficiency and A1C Levels Among Adults Without Diabetes in the National Health and Nutrition Examination Survey, 1999-2006. Diabetes Care. 2010 Apr 1;33(4):780–5.

11. Little RR, Roberts WL. A review of variant hemoglobins interfering with hemoglobin A1c measurement. J Diabetes Sci Technol. 2009 May 1;3(3):446–51.

12. Slama L, Palella FJ, Abraham AG, Li X, Vigouroux C, Pialoux G, et al. Inaccuracy of haemoglobin A1c among HIV-infected men: effects of CD4 cell count,

For peer review only - http://bmjopen.bmj.com/site/about/guidelines.xhtml

antiretroviral therapies and haematological parameters. J Antimicrob Chemother. 2014 Dec 1;69(12):3360–7.

13. Glesby MJ, Hoover DR, Shi Q, Danoff A, Howard A, Tien P, et al. Glycated haemoglobin in diabetic women with and without HIV infection: data from the Women's Interagency HIV Study. Antivir Ther. 2010;15(4):571–7.

14. Herman WH. Do Race and Ethnicity Impact Hemoglobin A1c Independent of Glycemia? J Diabetes Sci Technol. 2009 Jul;3(4):656–60.

15. Cavagnolli G, Pimentel AL, Freitas PAC, Gross JL, Camargo JL. Effect of ethnicity on HbA1c levels in individuals without diabetes: Systematic review and meta-analysis. PLoS One. 2017 Feb 13;12(2):e0171315.

16. Crampin AC, Kayuni N, Amberbir A, Musicha C, Koole O, Tafatatha T, et al. Hypertension and diabetes in Africa: design and implementation of a large population-based study of burden and risk factors in rural and urban Malawi. Emerg Themes Epidemiol. 2016 Dec;13(3).

17. Msyamboza KP, Mvula CJ, Kathyola D. Prevalence and correlates of diabetes mellitus in Malawi: population-based national NCD STEPS survey. BMC Endocr Disord. 2014 May 12;14:41.

18. Crampin AC, Dube A, Mboma S, Price A, Chihana M, Jahn A, et al. Profile: The Karonga Health and Demographic Surveillance System. Int J Epidemiol. 2012 Jun 1;41(3):676–85.

19. The Expert Committee on the Diagnosis and Classification of Diabetes Mellitus. Follow-up Report on the Diagnosis of Diabetes Mellitus. Diabetes Care. 2003 Nov 1;26(11):3160–7.

20. Youden WJ. Index for rating diagnostic tests. Cancer. 1950 Jan;3(1):32–5.

21. Mayega RW, Guwatudde D, Makumbi FE, Nakwagala FN, Peterson S, Tomson G, et al. Comparison of fasting plasma glucose and haemoglobin A1c point-of-care tests in screening for diabetes and abnormal glucose regulation in a rural low income setting. Diabetes Res Clin Pract. 2014 Apr;104(1):112–20.

22. Bertran EA, Berlie HD, Taylor A, Divine G, Jaber LA. Diagnostic performance of HbA_{1c} for diabetes in Arab vs. European populations: a systematic review and meta-analysis. Diabet Med. 2017 Feb;34(2):156–66.

23. Yan S, Liu S, Zhao Y, Zhang W, Sun X, Li J, et al. Diagnostic accuracy of HbA_{1c} in diabetes between Eastern and Western. Eur J Clin Invest. 2013 Jul;43(7):716–26.

24. Kodama S, Horikawa C, Fujihara K, Hirasawa R, Yachi Y, Yoshizawa S, et al. Use of high-normal levels of haemoglobin A1C and fasting plasma glucose for diabetes

For peer review only - http://bmjopen.bmj.com/site/about/guidelines.xhtml

screening and for prediction: a meta-analysis. Diabetes Metab Res Rev. 2013 Nov;29(8):680–92.

25. Hird TR, Pirie FJ, Esterhuizen TM, O'Leary B, McCarthy MI, Young EH, et al. Burden of Diabetes and First Evidence for the Utility of HbA1c for Diagnosis and Detection of Diabetes in Urban Black South Africans: The Durban Diabetes Study. Staiger H, editor. PLoS One. 2016 Aug 25;11(8):e0161966.

26. Zemlin AE, Matsha TE, Hassan MS, Erasmus RT. HbA1c of 6.5% to diagnose diabetes mellitus--does it work for us?--the Bellville South Africa study. PloS One. 2011;6(8):e22558.

For peer review only

For peer review only - http://bmjopen.bmj.com/site/about/guidelines.xhtml

Table 1. Demographic and clinical characteristics of participants in Karonga and Lilongwe, Malawi, 2013-2017.

	Total (n=3645) Median and IQR or n and %	Karonga (n=1613) Median and IQR, or n and %	Lilongwe (n=2032) Median and IQR, or n and %
Age, years	33 (25-44)	34 (26-47)	31 (24-41)
Female sex, %	63.0	58.3	67.2
Body mass index, kg/m^2	22.6 (20.5-26.0)	21.8 (20.0-23.8)	23.8 (21.2-28.0)
Normal BMI, %	61.1	81.9	57.8
Overweight BMI, %	20.4	13.8	26.1
Obese BMI, %	10.5	4.3	16.0
Systolic blood pressure, mm Hg	119 (111-129.5)	117.5 (110-127.5)	121 (112.5-131)
Diastolic blood pressure, mm Hg	72.5 (66.5-79.5)	72 (66-78)	73 (67-80.5)
Total cholesterol, mmol/l	3.8 (3.3-4.6)	3.8 (3.2-4.5)	3.9 (3.3-4.6)
Triglycerides, mmol/l	0.80 (0.58-1.14)	0.81 (0.58-1.12)	0.80 (0.59-1.18)
High density lipoprotein, mmol/l	1.12 (0.94-1.31)	1.09 (0.91-1.28)	1.15 (0.98-1.34)
Low density lipoprotein, mmol/l	2.53 (2.06-3.10)	2.44 (2.00-2.96)	2.62 (2.14-3.22)
Haemoglobin, g/dl	13.7 (12.6-14.9)	13.7 (12.6-15.0)	13.7 (12.6-14.9)
Normal haemoglobin, %	82.0	81.1	82.7
Anemia, %	18.0	18.9	17.3
HIV positive, %	8.5	7.8	9.1

For peer review only - http://bmjopen.bmj.com/site/about/guidelines.xhtml

HbA_{1c} %	5.3 (4.9-5.7)	5.1 (4.7-5.5)	5.4 (5.1-5.8)
$HbA_{1c} \geq 6.5\%$ (48 mmol/mol), %	7.3	5.6	8.8
Fasting plasma glucose, mmol/l	4.7 (4.3-5.0)	4.7 (4.3-5.0)	4.7 (4.4-5.1)
FPG 6.1-7.0 mmol/L, %	1.8	1.7	1.9
FPG ≥ 7.0, %	1.7	1.3	2.1

FPG, Fasting plasma glucose; IRQ, Interquartile range

Aside counts, the figures are re-weighted by inverse probability of receiving HbA_{1c} testing

For peer review only

For peer review only - http://bmjopen.bmj.com/site/about/guidelines.xhtml

Table 2. Sensitivity, specificity, and positive and negative likelihood ratios for detecting type 2 diabetes at selected HbA_{1c} thresholds for participants in Karonga and Lilongwe, Malawi, 2013-2017.

HbA_{1c} threshold			Sensitivity (%)	Specificity (%)	Positive Likelihood Ratio	Negative Likelihood Ratio
%	mmol/mol	SD				
6.0	42	+1.0	84.4	86.9	6.5	0.18
6.5*	48	+1.7	78.7	94.0	13.3	0.23
6.6†	49	+1.8	78.3	94.6	14.5	0.23
6.7	50	+2.0	76.5	96.0	19.0	0.25
7.4	57	+3.0	66.3	98.5	43.3	0.34
8.1	65	+4.0	54.8	99.5	117.4	0.45

SD, Standard Deviations from the mean HbA_{1c} score among diabetes-negative participants

* Threshold recommended by American Diabetes Association/European Association for the Study of Diabetes/International Diabetes Federation.

† Threshold identified by Youden's J statistic

Figures are re-weighted by the inverse probability of receiving HbA_{1c} testing

For peer review

For peer review only - http://bmjopen.bmj.com/site/about/guidelines.xhtml

Table 3. Overall and stratum-specific area under the receiver operating characteristic (AUROC) curves for detection of type 2 diabetes with HbA_{1c} among adults in Malawi, 2013-2017.

	AUROC (95% CI)	Sensitivity (%)*	Specificity (%)*	Positive Likelihood Ratio*	Negative Likelihood Ratio*
Overall	0.92 (0.90-0.94)	78.7	94.0	13.3	0.23
Site					
Karonga	0.88 (0.94-0.92)	70.2	95.4	15.2	0.31
Lilongwe	0.94 (0.92-0.96)	83.3	92.9	11.7	0.18
Sex					
Female	0.93 (0.91-0.95)	79.1	95.2	16.5	0.22
Male	0.89 (0.85-0.93)	77.6	92.1	9.9	0.24
Body mass index					
Normal	0.87 (0.83-0.92)	70.1	95.1	14.5	0.31
Overweight	0.93 (0.89-0.96)	84.5	91.4	9.9	0.17
Obese	0.92 (0.89-0.95)	80.4	90.6	8.6	0.22
Haemoglobin					
Normal	0.93 (0.91-0.95)	81.4	93.5	12.6	0.20
Anemia	0.86 (0.79-0.93)	61.5	96.4	17.4	0.40

* At $HbA_{1c} \geq 6.5\%$

Figures are re-weighted by the inverse probability of receiving HbA_{1c} testing

For peer review only

1 Table 4. Clinical characteristics of participants identified as having diabetes by fasting
2 blood glucose, and for true- and false-positive participants by HbA_{1c}≥6.5% in Karonga
3 and Lilongwe, Malawi, 2013-2017.

	Type 2 diabetes positive by FPG ≥7.0 mmol/L (IQR)	Type 2 diabetes true positive by HbA_{1c}≥6.5%* (IQR)	Type 2 diabetes false positive by HbA_{1c}≥6.5%*† (IQR)
Age, years	50 (41-60)	51 (43-61)	35 (23-51)
Female sex, %	65.6	66.3	51.0
Body mass index, kg/m^2	27.9 (24.2-32.5)	28.2 (24.8-32.5)	24.8 (20.9-28.3)
Systolic blood pressure, mm Hg	133.5 (121.5-153)	135 (123-156)	125 (115-136)
Diastolic blood pressure, mm Hg	81.5 (74.5-89.5)	81.5 (75.0-90.5)	74.5 (68.5-82.5)
Total cholesterol, mmol/L	4.9 (4.1-5.7)	5.0 (4.3-5.8)	4.2 (3.5-5.2)
Triglycerides, mmol/L	1.7 (1.2-2.3)	1.8 (1.2-2.5)	0.9 (0.6-1.5)
High density lipoprotein, mmol/L	1.04 (0.89-1.22)	1.03 (0.88-1.20)	1.15 (0.97-1.29)
Low density lipoprotein, mmol/L	3.38 (2.69-4.09)	3.52 (2.89-4.22)	2.82 (2.36-3.56)
Haemoglobin, g/dl	13.9 (12.9-14.9)	13.9 (12.9-15.0)	14.2 (13.1-15.1)
HbA_{1c}, %	8.4 (6.9-11.3)	9.6 (7.7-11.8)	6.9 (6.6-7.4)

For peer review only - http://bmjopen.bmj.com/site/about/guidelines.xhtml

Fasting plasma glucose, mmol/L	9.5 (7.7-14.2)	10.7 (8.3-15.4)	4.8 (4.5-5.1)

FPG, Fasting Plasma Glucose; IQR, Interquartile range

* Threshold recommended by American Diabetes Association, European Association for the Study of Diabetes, and International Diabetes Federation.

Aside counts, figures are weighted by the inverse probability of receiving HbA_{1c} testing

For peer review only

For peer review only - http://bmjopen.bmj.com/site/about/guidelines.xhtml

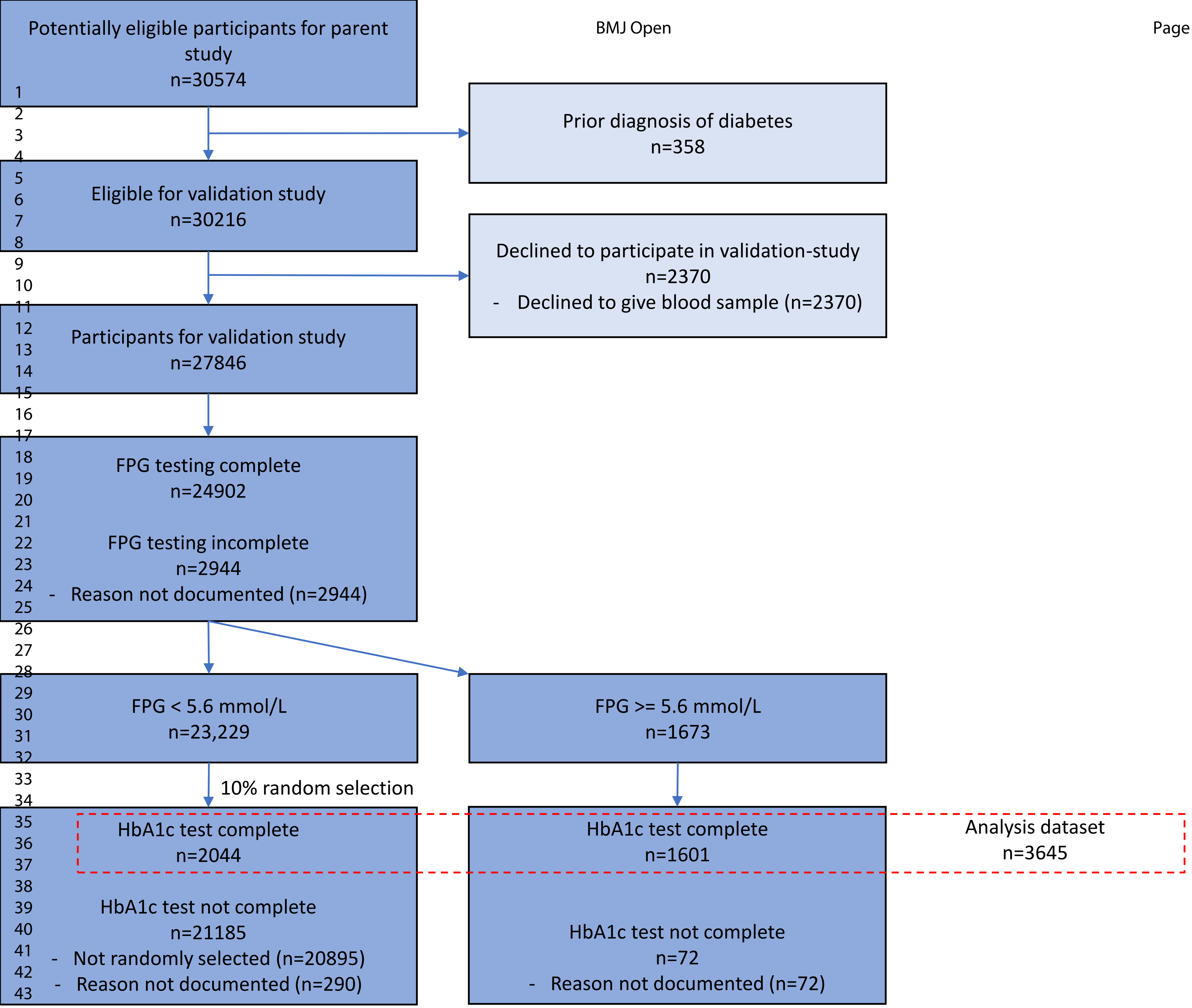
Potentially eligible participants for parent study
n=30574
Prior diagnosis of diabetes
n=358
Eligible for validation study
n=30216
Declined to participate in validation-study
n=2370
- Declined to give blood sample (n=2370)
Participants for validation study
n=27846
FPG testing complete
n=24902
FPG testing incomplete
n=2944
- Reason not documented (n=2944)
FPG < 5.6 mmol/L
n=23,229
FPG >= 5.6 mmol/L
n=1673
10% random selection
HbA1c test complete
n=2044
HbA1c test not complete
n=21185
- Not randomly selected (n=20895)
- Reason not documented (n=290)
HbA1c test complete
n=1601
HbA1c test not complete
n=72
- Reason not documented (n=72)
Analysis dataset
n=3645

```
*******************
*** Hb1Ac AUROC ***
*******************
          use "30Aug17.dta", clear
          keep gluc_units hba1_units ///
                    BMI systolic diastolic chol_units tg_units hdl ldl hgb ///
                    sex site stid age
          qui compress
          sum

****************
*** NEW VARS ***
*****************************************************************
          gen diabetes_fpg=(gluc_units>=7.0)
          label variable diabetes_fpg "Newly diabetes positive (fasting plasma glucose>=7)"
          label define yesno 0 No 1 Yes
          label values diabetes_fpg yesno

          recode gluc_units (min/6.09=0)(6.10/6.99=1)(7.0/max=0), gen(prediabetes)
          label variable prediabetes "Pre-diabetes (fasting glucose of 6.1-6.9)"
          label values prediabetes yesno

          recode gluc_units (min/5.59=0)(5.6/max=1), gen(gluc56)
          label variable gluc56 "Fasting glucose >=5.6 mmol/L"
          label values gluc56 yesno

          recode hba1 (min/6.49=0)(6.50/max=1), gen(hba65)
          label variable hba65 "Diabetes screen positive (HbA1c >= 6.5%)"
          label values hba65 yesno

          recode hba1 (min/6.79=0)(6.80/max=1), gen(hba68)
          label variable hba68 "Diabetes screen pos (HbA1c >=6.8%)"
          label values hba68 yesno

          gen hbaint = round(hba1_units,1)
          label variable hbaint "HbA1c score, rounded to integer"

          gen hbadec = round(hba1_units,0.1)
          label variable hbaint "HbA1c score, rounded to tenths"

          encode sex, gen(gender)
          label define gender 1 Female 2 Male, modify
          label values gender gender
          label variable gender "Sex"
          drop sex
          rename gender sex

          gen tempsite = site=="L"
          label define site 0 Karonga 1 Lilongwe, modify
          label values tempsite site
          drop site
          rename tempsite site
          label variable site "Research site"

          gen hbatest = !mi(hba1)
          gen pw =!mi(hba1)
          recode pw (1=10) if gluc_units<5.6
```

For peer review only

```
        label variable pw "Probability sampling weight for HbA1c testing"

        sort stid
        gen pid = _n
        label variable pid "Unique identifier"
        expand=pw
        summarize hba1_unit if diabetes_fpg==0
        gen meanhba = r(mean)
        gen sdhba = r(sd)
        gen zscore = (hba1_units - meanhba) / sdhba
        label variable zscore "HbA1c z-score"
        notes zscore: Using only HbA1c scores from diabetes-negative pts and
re-weighted for the sub-sampling
        duplicates drop pid, force

        gen zdec = round(zscore, 0.1)
        label variable zdec "Normal HbA1c z-score, rounded to 0.1"

        recode BMI (min/24.9999=0)(25/29.999=1)(30/max=2), gen(bmi_3c)
        label define bmi 0 Normal 1 Overweight 2 Obese
        label values bmi_3c bmi
        label variable bmi_3c "BMI category"

        gen anemia=0
        replace anemia=1 if sex==2 & hgb<13.5
        replace anemia=1 if sex==1 & hgb<12.0
        label values anemia yesno

        quiet compress

        tempfile full
        save `full'
*** END NEW VARS ***********************************************

*** FLOW CHART ***
        use `full', clear
        count
        drop if mi(gluc_units)
        drop if mi(hba1)
        tab gluc56, sum(hba1)

**************************
*** DESCRIPTIVE CHECKS ***
**************************
        sum age sex site hba1 gluc_units diabetes_fpg pw          /* Most of
these are skewed */
        logistic diabetes_fpg hba1_units [pw=pw]
        prop diabetes_fpg
        prop diabetes_fpg [pw=pw]


****************************************
*** TABLE 1: Patient characteristics ***
****************************************
        tabstat age sex BMI syst dias chol_units tg_units hdl_units
ldl_units hgb hba1_units gluc_units[aw=pw], stat(n p50 p25 p75) by(site)
f(%9.2f) col(s)
        foreach var of varlist sex bmi_3c anemia hba65 prediabetes
diabetes_fpg {
                prop `var' [pw=pw]
```

For peer review only

```
                    prop `var' [pw=pw], over(site)
                    }
          replace prediabetes=. if gluc_units>=7.0

********************************
*** TABLE 2: Validity stats ***
********************************
          * Overall dataset for HbA1c vs diabetes
          foreach x of varlist diabetes_fpg prediabetes {
                    somersd `x' hba1 [pw=pw], trans(c)
                    roctab `x' hba1, binomial sum
                    senspec `x' hbadec [pw=pw], sensitivity(sens)
specificity(spec) ntpos(tp) nfpos(fp) ntneg(tn) nfneg(fn)
                    for var sens spec tp fp tn fn: replace X = 100*X
                    gen ppv = 100*tp / (tp+fp)
                    gen npv = 100*tn / (tn+fn)
                    gen lrpos = sens / (100-spec)
                    gen lrneg = (100-sens) / spec
                    gen jtemp = sens + spec - 100
                    egen youdenj = max(jtemp)
                    replace youdenj=. if youdenj!=jtemp
                    table zdec, c(mean sens mean spec mean youdenj mean lrpos
mean lrneg) format(%9.2f)
                    drop sens spec tp fp tn fn ppv npv lrpos lrneg jtemp
youdenj
          }

***************
*** TABLE 3 ***
***************
          * Stratum-specific HbA1c vs diabetes
          foreach var of varlist site sex bmi_3c anemia {
                    levelsof `var', local(levels)
                    foreach l of local levels {
                              tab `var' if `var'==`l'
                              somersd diabetes_fpg hba1 [pw=pw] if `var'==`l',
trans(c)
                              senspec diabetes_fpg hbadec [pw=pw] if
`var'==`l', sensitivity(sens) specificity(spec)
                              for var sens spec: replace X = 100*X
                              gen lrpos = sens / (100-spec)
                              gen lrneg = (100-sens) / spec
                              table zdec if zdec>1.66 & zdec<1.71 & `var'==`l',
c(mean sens mean spec mean lrpos mean lrneg) format(%9.2f)
                              drop sens spec lrpos lrneg
                              }
                    }

********************************
*** Table 4: Diabetes chars ***
********************************
* Present clinical charteristics of diabetes+ by fasting glucose criteria
          tabstat age sex BMI syst dias chol_units tg_units hdl_units
ldl_units hgb hba1_units gluc_units  if diabetes_fpg [aw=pw], stat(p50 p25
p75) f(%9.2f) col(s)
          prop sex [pw=pw] if diabetes_fpg

* Present clinical characteristics among HbA1c+, by true status
          tab hba65 diabetes_fpg
          tabstat age sex BMI syst dias chol_units tg_units hdl_units
```

For peer review only

```
ldl_units hgb hba1_units gluc_units  if hba65 [aw=pw], by(diabetes_fpg)
stat(p50 p25 p75) f(%9.2f) col(s)
          prop sex if hba65 [pw=pw], over(diabetes_fpg)
```

For peer review only

Section & Topic	No	Item	Reported on page #
**TITLE OR ABSTRACT**			
	**1**	Identification as a study of diagnostic accuracy using at least one measure of accuracy (such as sensitivity, specificity, predictive values, or AUC)	1
**ABSTRACT**			
	**2**	Structured summary of study design, methods, results, and conclusions (for specific guidance, see STARD for Abstracts)	2
**INTRODUCTION**			
	**3**	Scientific and clinical background, including the intended use and clinical role of the index test	4
	**4**	Study objectives and hypotheses	5
**METHODS**			
Study design	**5**	Whether data collection was planned before the index test and reference standard were performed (prospective study) or after (retrospective study)	5
Participants	**6**	Eligibility criteria	5
	**7**	On what basis potentially eligible participants were identified (such as symptoms, results from previous tests, inclusion in registry)	5
	**8**	Where and when potentially eligible participants were identified (setting, location and dates)	5
	**9**	Whether participants formed a consecutive, random or convenience series	5
Test methods	**10a**	Index test, in sufficient detail to allow replication	5-6
	**10b**	Reference standard, in sufficient detail to allow replication	5-6
	**11**	Rationale for choosing the reference standard (if alternatives exist)	
	**12a**	Definition of and rationale for test positivity cut-offs or result categories of the index test, distinguishing pre-specified from exploratory	6-7
	**12b**	Definition of and rationale for test positivity cut-offs or result categories of the reference standard, distinguishing pre-specified from exploratory	6
	**13a**	Whether clinical information and reference standard results were available to the performers/readers of the index test	6
	**13b**	Whether clinical information and index test results were available to the assessors of the reference standard	6
Analysis	**14**	Methods for estimating or comparing measures of diagnostic accuracy	6
	**15**	How indeterminate index test or reference standard results were handled	7
	**16**	How missing data on the index test and reference standard were handled	7
	**17**	Any analyses of variability in diagnostic accuracy, distinguishing pre-specified from exploratory	NA
	**18**	Intended sample size and how it was determined	8
**RESULTS**			
Participants	**19**	Flow of participants, using a diagram	Figure 1
	**20**	Baseline demographic and clinical characteristics of participants	Table 1
	**21a**	Distribution of severity of disease in those with the target condition	Table 4
	**21b**	Distribution of alternative diagnoses in those without the target condition	NA
	**22**	Time interval and any clinical interventions between index test and reference standard	6
Test results	**23**	Cross tabulation of the index test results (or their distribution) by the results of the reference standard	9
	**24**	Estimates of diagnostic accuracy and their precision (such as 95% confidence intervals)	9
	**25**	Any adverse events from performing the index test or the reference standard	8
**DISCUSSION**			
	**26**	Study limitations, including sources of potential bias, statistical uncertainty, and generalisability	12
	**27**	Implications for practice, including the intended use and clinical role of the index test	12
**OTHER INFORMATION**			
	**28**	Registration number and name of registry	NA
	**29**	Where the full study protocol can be accessed	NA
	**30**	Sources of funding and other support; role of funders	12

For peer review only - http://bmjopen.bmj.com/site/about/guidelines.xhtml

STARD 2015

AIM

STARD stands for "Standards for Reporting Diagnostic accuracy studies". This list of items was developed to contribute to the completeness and transparency of reporting of diagnostic accuracy studies. Authors can use the list to write informative study reports. Editors and peer-reviewers can use it to evaluate whether the information has been included in manuscripts submitted for publication.

EXPLANATION

A **diagnostic accuracy study** evaluates the ability of one or more medical tests to correctly classify study participants as having a **target condition.** This can be a disease, a disease stage, response or benefit from therapy, or an event or condition in the future. A medical test can be an imaging procedure, a laboratory test, elements from history and physical examination, a combination of these, or any other method for collecting information about the current health status of a patient.

The test whose accuracy is evaluated is called **index test.** A study can evaluate the accuracy of one or more index tests. Evaluating the ability of a medical test to correctly classify patients is typically done by comparing the distribution of the index test results with those of the **reference standard**. The reference standard is the best available method for establishing the presence or absence of the target condition. An accuracy study can rely on one or more reference standards.

If test results are categorized as either positive or negative, the cross tabulation of the index test results against those of the reference standard can be used to estimate the **sensitivity** of the index test (the proportion of participants *with* the target condition who have a positive index test), and its **specificity** (the proportion *without* the target condition who have a negative index test). From this cross tabulation (sometimes referred to as the contingency or "2x2" table), several other accuracy statistics can be estimated, such as the positive and negative **predictive values** of the test. Confidence intervals around estimates of accuracy can then be calculated to quantify the statistical **precision** of the measurements.

If the index test results can take more than two values, categorization of test results as positive or negative requires a **test positivity cut-off**. When multiple such cut-offs can be defined, authors can report a receiver operating characteristic (ROC) curve which graphically represents the combination of sensitivity and specificity for each possible test positivity cut-off. The **area under the ROC curve** informs in a single numerical value about the overall diagnostic accuracy of the index test.

The **intended use** of a medical test can be diagnosis, screening, staging, monitoring, surveillance, prediction or prognosis. The **clinical role** of a test explains its position relative to existing tests in the clinical pathway. A replacement test, for example, replaces an existing test. A triage test is used before an existing test; an add-on test is used after an existing test.

Besides diagnostic accuracy, several other outcomes and statistics may be relevant in the evaluation of medical tests. Medical tests can also be used to classify patients for purposes other than diagnosis, such as staging or prognosis. The STARD list was not explicitly developed for these other outcomes, statistics, and study types, although most STARD items would still apply.

DEVELOPMENT

This STARD list was released in 2015. The 30 items were identified by an international expert group of methodologists, researchers, and editors. The guiding principle in the development of STARD was to select items that, when reported, would help readers to judge the potential for bias in the study, to appraise the applicability of the study findings and the validity of conclusions and recommendations. The list represents an update of the first version, which was published in 2003.

More information can be found on http://www.equator-network.org/reporting-guidelines/stard.

For peer review only

CC BY SA

Glycated hemoglobin A1c (HbA1c) for detection of diabetes mellitus and impaired fasting glucose in Malawi: a diagnostic accuracy study

Journal:	*BMJ Open*
Manuscript ID	bmjopen-2017-020972.R1
Article Type:	Research
Date Submitted by the Author:	23-Feb-2018
Complete List of Authors:	Rathod, Sujit; London School of Hygiene and Tropical Medicine, Department of Population Health<br>Crampin, Amelia; London School of Hygiene and Tropical Medicine<br>Musicha, Crispin; Malawi Epidemiology and Intervention Research Unit<br>Kayuni, Ndoliwe; Malawi Epidemiology and Intervention Research Unit<br>Banda, Louis; Malawi Epidemiology and Intervention Research Unit<br>Saul, Jacqueline; London School of Hygiene and Tropical Medicine<br>McLean, Estelle; London School of Hygiene and Tropical Medicine; Malawi Epidemiology and Intervention Research Unit<br>Branson, Keith; London School of Hygiene and Tropical Medicine<br>Jaffar, Shabbar; Liverpool School of Tropical Medicine<br>Nyirenda, Moffat; London School of Hygiene and Tropical Medicine
**Primary Subject Heading**:	Diabetes and endocrinology
Secondary Subject Heading:	Diagnostics
Keywords:	HbA1c, Type 2 diabetes, Malawi, validity, receiver operating characteristic

SCHOLARONE™
Manuscripts

Glycated hemoglobin A_{1c} (HbA_{1c}) for detection of diabetes mellitus and impaired fasting glucose in Malawi: a diagnostic accuracy study

Sujit D Rathod*,[1] Amelia C Crampin,[1] Crispin Musicha,[2] Ndoliwe Kayuni,[2]

Louis Banda,[2] Jacqueline Saul,[1] Estelle McLean,[1,2] Keith Branson,[1] Shabbar Jaffar,[3] Moffat J Nyirenda,[1]

[1] London School of Hygiene & Tropical Medicine, London, United Kingdom

[2] Malawi Epidemiology and Intervention Research Unit, Chilumba, Malawi

[3] Liverpool School of Tropical Medicine, Liverpool, United Kingdom

*Corresponding author

Department of Population Health

London School of Hygiene & Tropical Medicine

Keppel Street

London WC1E7HT

United Kingdom

For peer review only

P: +44 020 7299 4683

F: +44 (0)20 7436 4230

sujit.rathod@lshtm.ac.uk

For peer review only

Abstract

Objectives: To examine the accuracy of glycated hemoglobin A_{1c} (HbA_{1c}) in detecting type 2 diabetes and impaired fasting glucose among adults living in Malawi.

Design: A diagnostic validation study of HbA_{1c}. Fasting plasma glucose (FPG) ≥7.0 mmol/L was the reference standard for Type 2 diabetes, and FPG between 6.1 and 6.9 mmol/L as impaired fasting glucose.

Participants: 3645 adults (of whom 63% female) recruited from two demographic surveillance study sites in urban and rural Malawi. This analysis excluded those who had a previous diagnosis of diabetes or had history of taking diabetes medication.

Results: HbA_{1c} demonstrated excellent validity to detect FPG-defined diabetes, with an area under the receiver operating characteristic (AUROC) curve of 0.92 (95% CI 0.90-0.94). At HbA_{1c}≥6.5% (48 mmol/mol), sensitivity was 78.7% and specificity was 94.0%. Subgroup AUROCs ranged from 0.86 for participants with anaemia to 0.94 for participants in urban Malawi. There were clinical and metabolic differences between participants who were true diabetes vs. false positives when HbA_{1c}≥6.5% (48 mmol/mol).

Conclusions: The findings from this study provide justification to use HbA_{1c} to detect type 2 diabetes. As HbA_{1c} testing is substantially less burdensome to patients than either fasting plasma glucose testing or oral glucose tolerance testing, it represents a useful option for expanding access to diabetes care in sub-Saharan Africa.

For peer review only

Strengths and limitations of this study

- Only two studies have assessed the validity of HbA_{1c} to detect type 2 diabetes in African-origin populations; this is the first such study from Malawi.
- We used fasting plasma glucose as the reference standard, which is commonly used in clinical practice and for validation studies.
- The results of this study may not be generalizable to other populations in sub-Saharan Africa, or those with anaemia.

For peer review only

Over 400 million people live with type 2 diabetes worldwide, with more than 75% of these in low- and middle-income countries (1). While the prevalence of diabetes in sub-Saharan African is lower than in other global regions, around 6-9% (2,3), demographic models project that this region will experience the fastest growth rate in cases over the next 20 years (1). Approximately 50-66% of people with diabetes are undiagnosed (1,4), and complications are common even after diagnosis (5). As barriers to diabetes care become surmounted in this region (4), focus must turn to improving the accessibility of diagnostics.

The diagnosis of diabetes has traditionally been based upon the detection of elevated plasma glucose levels, either after fasting or two hours after an oral glucose tolerance test, or, in symptomatic individuals, after a random blood glucose check (6). While these tests are available in sub-Saharan Africa, access to services are limited, travel times and clinic waiting times are high, and integrity of the samples and quality of the measuring tools are in most cases uncertain. More reliable approaches to circumvent some of these challenges are required. Recently, the American Diabetes Association (ADA) and the World Health Organization (WHO) have recommended using glycated hemoglobin A_{1c} (HbA_{1c}) in blood to diagnose diabetes mellitus (7,8). HbA_{1c} testing provides significant practical advantages over glucose testing as it does not fluctuate appreciably and thus can be performed at any time of the day. Further, HbA_{1c} testing does not require any special pre-test preparations, such as overnight fasting or glucose loading.

There are acknowledged limitations when attempting to measure HbA_{1c} levels which may be relevant for diagnostics in sub-Saharan Africa (6,8,9). It is unclear whether one can use HbA_{1c} to diagnose diabetes for people who have malaria (6), haemolytic anaemia

For peer review only

(10,11), sickle cell anaemia (12), HIV infection (13,14) or who are of African descent (15,16). Given uncertainty around the validity of HbA_{1c} to diagnose type 2 diabetes and its precursor, impaired fasting glucose, in sub-Saharan Africa, we conducted a diagnostic accuracy study in Malawi, and have reported results here.

Design and Methods

Study setting

This report uses data collected as part of a Malawi Epidemiology and Intervention Research Unit (MEIRU) survey, which aimed to understand the burden and risk factors of non-communicable diseases in Malawi (17), where the national prevalence of diabetes is estimated to be 5.6% (18). Community-based cross-sectional surveys were conducted in Karonga District (May 2013-April 2016) (19) and Lilongwe City (June 2013-April 2017) (17). Karonga is a rural, low-altitude, malaria-endemic district in northern Malawi, and Lilongwe is an urban, high altitude city in central Malawi with lower malaria prevalence.

Recruitment and data collection

Detailed study procedures have been previously reported (17). All adults aged 18 years and above who were usually resident in either of the study sites were eligible to participate in the parent study. All households were approached consecutively, and all residents aged ≥18 years were recruited. Recruits provided written informed consent for each separate study component (i.e. standardized interview, physical measurements and blood specimen collection) and could opt out of any component. Venipuncture was

For peer review only

conducted after a minimum 8-hour fasting period and whole blood samples were collected in sodium fluoride tubes. Tubes were stored on ice in an insulated cool box and delivered to the laboratory for processing (mean delivery time of 2.6 hours after blood collection), and glucose analysis was completed within 1 hour after processing. Quantitative determination of fasting plasma glucose (hexokinase method) and HbA_{1c} was performed using the Beckman Coulter Chemistry Analyzer AU480 system according to the manufacturer's guidelines.

FPG testing was conducted on all participant samples. HbA_{1c} testing was conducted on all participants samples for which FPG results were greater than 5.6 mmol/L and in a 10% random sample of those with FPG lower than 5.6 mmol/L. The lab technician who conducted the HbA_{1c} test was blind to the participants' clinical characteristics and FPG result. Nearly all (87%) of HbA_{1c} tests were completed within 24 hours of FPG test. Cut-points recommended by WHO were used to define fasting plasma glucose (FPG) as normal (<6.1 mmol/L), impaired fasting glucose (6.1 to 6.9 mmol/L) or diabetes (≥7.0 mmol/L) (20). Categories of body mass index (BMI) for underweight/normal ($BMI<25kg/m^2$), overweight ($25\leq BMI<30$) and obese ($BMI\geq30$), and categories for anaemia (haemoglobin<12.0g/dL for women and <13.5 for men) were used.

Statistical analyses

Participants who reported a past diagnosis of diabetes or history of taking diabetes medication were excluded from this analysis. First, demographic and clinical variables (i.e. age, sex, location, BMI, blood pressure, plasma glucose and lipid profile, and HIV serostatus) were described separately for the Karonga and Lilongwe samples. Medians

For peer review only

and interquartile ranges were reported for the continuous measures, as most of these measures were skewed, and proportions for categorical measures. Second, distribution of HbA_{1c} level was compared by FPG result (<7.0 vs ≥7.0 mmol/L) with logistic regression. Third, the validity of HbA_{1c} for diagnosing diabetes, using FPG (>7.0 mmol/L) as reference standard, was assessed by using Somers' D statistic with Harrell's C transformation to estimate the area under the receiver operating characteristics (AUROC) curve and 95% confidence interval. Validity statistics (i.e. sensitivity, specificity, and positive and negative likelihood ratios) were reported for standardized HbA_{1c} scores which correspond to 1.0, 2.0, 3.0 and 4.0 standard deviations above the sample's mean HbA_{1c} score, at an 'optimal' value identified by the Youden's J statistic (where the sum of the sensitivity and specificity reaches its maximum) (21), and at 6.5% (48 mmol/mol), which is the value recommended by several diabetes associations. The standardized values were generated using the distribution of HbA_{1c} scores from the diabetes-negative participants with HbA_{1c} test results (i.e. all participants with fasting plasma glucose ≥5.6 mmol/L and a 10% sub-sample of participants with plasma glucose <5.6) such that the sub-sampled participants were upweighted 10-fold before generating the distribution. Fourth, to consider the validity of HbA_{1c} to detect diabetes across subgroups, the ROC analysis was stratified by site, sex, BMI, and hemoglobin level. Within each stratum the sensitivity, specificity, positive and negative likelihood ratio when using HbA_{1c}≥6.5% (48 mmol/mol) as a cut off score were reported. Fifth, the AUROC and 95% CI for detecting impaired fasting glucose (FPG ≥ 6.1 mmol/L) with HbA_{1c} was estimated among those who did not have diabetes (FPG <7.0 mmol/L). Finally, the clinical characteristics were described for the subsets of participants who had FPG-defined diabetes, and for

For peer review only

true- and false-positive participants with $HbA_{1c} \geq 6.5\%$ (48 mmol/mol). Again, medians and interquartile ranges were reported for continuous measures and proportions for categorical measures. The analysis was conducted using Stata SE 14.2 (StataCorp, College Station, Texas, USA)(See Stata code in supplemental material) with complete-case analysis. Aside count figures, the statistical results were weighted by the inverse probability of receiving the HbA_{1c} test.

Ethical approval

The study protocol was reviewed and approved by the Malawi National Health Sciences Research Committee, and London School of Hygiene and Tropical Medicine (UK) Ethics Committee. Participants identified as having diabetes were referred to chronic care clinics established in conjunction with the Malawi Ministry of Health, where they initiated management per national treatment guidelines.

Patient and public involvement

How was the development of the research question and outcome measures informed by patients' priorities, experience, and preferences?

Our research is in line with the Malawi National Health Research Agenda. which recently prioritised non-communicable disease, and was undertaken in partnership with the Malawi National Ministry of Health and Malawi College of Medicine. Our community surveys that contributed to the work described in this paper followed extensive engagement with the community and policy makers to ensure that their views were represented.

For peer review only

How did you involve patients in the design of this study?

This was a community-based population study. Two demographic surveillance systems were designed to enroll all adults in the enumeration areas, regardless of health status. To ensure that the research adequately represented the needs of the population, including potential patients, the design and aims of the study were developed in a stakeholders' meeting in Lilongwe, the capital city of Malawi, attended by policy makers, policy implementers and researchers.

Were patients involved in the recruitment to and conduct of the study?

Village elders ("ndunas") and other responsible community members were recruited as key informants in the demographic surveillance systems. They key informants were responsible for enumerating households, and for reporting household vital events and household migrations. Prior to launching new studies, the research team conducts community sensitization events with dancing, dramas and question and answer sessions.

How will the results be disseminated to study participants?

Key informants are regularly brought to project headquarters for briefings about research procedures (e.g. specimen collection, laboratory testing, data storage), presentations of latest research findings, and discussion of how they will feed these findings back to the community.

For randomised controlled trials, was the burden of the intervention assessed by patients themselves?

For peer review only

Not applicable.

Results

As shown in the STARD flow diagram (Figure 1), 30,574 adults consented to the interview and clinical examination in the parent study, of whom 27,846 also provided consent for blood specimen collection, as was required for this sub-study. No participant experienced a severe adverse event due to the specimen collection. This analysis includes the 3645 participants (1613 in Karonga and 2032 in Lilongwe) who had both FPG and HbA_{1c} levels measured, and who did not report prior diagnosis of diabetes by a medical professional.

Demographic and Clinical Characteristics

The demographic and clinical characteristics of analysis participants are reported in Table 1.

For the 3645 participants, the median age was 33 years (IQR 24-44) and 63% were female. The median HbA_{1c} level was 5.3% (IQR 4.9-5.7) and 7.3% had HbA_{1c} level of 6.5% (48 mmol/mol) or higher. The median FPG level was 4.7 mmol/L (IQR 4.3-5.0); 1.8% of participants had a FPG level consistent with impaired fasting glucose and another 1.7% with diabetes.

Diagnostic validity of HbA_{1c}

For every percentage unit increase in HbA_{1c} score, there was an almost 3-fold increase in the odds of having FPG-defined diabetes (OR 2.80, 95% CI 2.21-3.53, R^2 0.371, Wald

For peer review only

$X^2(1)$ 75.5, P<0.001). Further, HbA_{1c} demonstrated excellent validity to detect for FPG-defined diabetes, with an AUROC of 0.92 (95% CI 0.90-0.94). Validity statistics are presented for a range of HbA_{1c} cut points in Table 2.

An HbA_{1c} value of 6.0% corresponded to 1.0 standard deviation above the mean in the diabetes-negative sample's distribution of HbA_{1c} scores; at this cut point 84.4% of people with diabetes would test positive (sensitivity) and 86.9% of people without diabetes would test negative (specificity). At the commonly-recommended HbA_{1c} threshold of 6.5% (48 mmol/mol), sensitivity was 78.7% and specificity was 94.0%. Youden's J was at an HbA_{1c} value of 6.6%, where sensitivity was 78.3% and specificity was 94.6%. Stratum-specific AUROCs (Table 3), ranged from a minimum of 0.86 for participants with anaemia to a maximum of 0.94 for participants in Lilongwe. With $HbA_{1c} \geq 6.5\%$ (48 mmol/mol), participants with anaemia had 61.5% sensitivity, 96.4% specificity and a positive likelihood ratio of 17.4, and the corresponding figures for participants in Lilongwe were 70.2%, 95.4%, and 15.2.

Among participants who did not have FPG-defined diabetes, the HbA_{1c} score had average validity to detect impaired fasting glucose (AUROC 0.70, 95% CI 0.67-0.73).

Clinical characteristics of participants meeting diagnostic criteria for diabetes

The clinical characteristics of participants who had FPG-defined diabetes, and for participants who had true positive and false positives results are presented in Table 4.

For peer review only

The participants who had FPG-defined diabetes (FPG ≥7.0 mmol/L) had a median age of 50 years (IQR 41-60) and 65.5% were female. The median BMI was 27.9 kg/m^2 (IQR 24.2-32.5) and the median systolic blood pressure 133.5 mm Hg (IQR 121.5-153). The characteristics of true positive participants (HbA_{1c} ≥6.5% (48 mmol/mol) and FPG≥7.0 mmol/L) and the false positive participants (HbA_{1c} ≥6.5% (48 mmol/mol) and FPG< 7.0 mmol/L) are also described in Table 4..

Discussion

In a large, multi-site sample of adults in Malawi, we found that HbA_{1c} is highly predictive of FPG-defined type 2 diabetes, a relationship which was consistent across several sub-groups. HbA_{1c} was less predictive for impaired fasting glucose. Using a cut off value of HbA_{1c} 6.5% (48 mmol/mol) to detect diabetes, there were demographic and clinical differences between true- and false-positive participants.

This analysis is one of the first to compare HbA_{1c} as a stand-alone test to detect type 2 diabetes in a black African population in sub-Saharan Africa, and the first from Malawi. In Uganda, Mayega et al found that HbA_{1c} had moderate validity (AUROC 0.75) for detecting FPG-defined diabetes (22), which was lower than found by Hird et al in South Africa (AUROC 0.95). These findings from sub-Saharan Africa complement meta-analyses of studies in East Asia, Middle East and Europe which show that HbA_{1c} is a valid test for detecting diabetes (23–25) across different ethnic groups.

There were several notable demographic and clinical differences between participants who were true vs false positives for FPG-defined diabetes at HbA_{1c}≥6.5% (48 mmol/mol). Compared to participants who had HbA_{1c}≥6.5% (48 mmol/mol) and

For peer review only

FPG<7.0 mmol/L, those who had both FPG≥7.0 and HbA_{1c}≥6.5% (48 mmol/mol) were more likely to be female, to be older, and to have higher BMI and blood pressure. These findings are consistent with data from the Uganda where the AUROC for all participants was 0.75, but was significantly higher at 0.90 for overweight participants and 0.98 for obese participants. This suggests that HbA_{1c} is particularly useful in in detecting diabetes in individuals with insulin resistance or metabolic syndrome (e.g. constellation of high BMI, hypertension, dyslipidemia). In contrast, the false positive group (HbA_{1c}≥6.5% (48 mmol/mol) and FPG<7.0 mmol/L) lacked features of metabolic syndrome. The false positive group is likely to include individuals with isolated postprandial hyperglycemia, rather than fasting hyperglycemia, (perhaps from pancreatic deficiency rather than insulin resistance) which would require oral glucose tolerance testing (OGTT) to demonstrate. Notably, HbA_{1c} has been shown to strongly predict OGTT-defined diabetes in South Africa, where Hird et al estimated the AUROC at 0.94 (26). The clinical and demographic differences between true- and false- positive participants observed here, though interesting, remain descriptive and will require confirmation in larger studies.

We found that HbA_{1c} had poor predictive ability for detecting impaired fasting glucose. This is consistent with other studies in Africa and evidence from a recent meta-analysis by Kodama et al (22,25,26). Accordingly, evidence strongly suggests that HbA_{1c} is not appropriate for detecting impaired fasting glucose.

Stratum-specific AUROC analysis indicated largely consistent values across strata (i.e. by sex, site, and BMI), which is evidence of suitability of HbA_{1c} to detect diabetes across a range of groups. However, AUROC value were lowest for those with anaemia – which appears to be driven by a drop in sensitivity. Different forms of anaemia (e.g. chronic or

For peer review only

acute) affect the integrity and quantity of HbA1c-carrying red blood cells, resulting in HbA_{1c} having diminished utility as a stand-alone diagnostic tool among individuals with anaemia (11). For example, Son et al also found that the AUROC for HbA_{1c} to detect OGTT-defined diabetes in South Korea was lower for adults with anaemia (0.86 vs 0.88) (10). Future research will be required to determine how to control anaemia in sub-Saharan Africa so that HbA_{1c} testing maintains its validity.

We used FPG rather than OGTT as the reference standard for diabetes in this analysis. A 2013 meta-analysis of 13 cohort studies found that FPG is highly correlated to present and to future diabetes (25), though none of the studies identified were from sub-Saharan Africa. A 2015 meta-analysis of diabetes measures found 27 validation studies of HbA_{1c} using FPG-defined diabetes as the diagnostic standard, compared to 9 using OGTT (27). FPG is typically preferred for validation studies due to its practical advantages, though it is unclear with proportion of individuals with diabetes are missed when FPG testing is not paired with OGTT. While FPG is highly correlated to present and to future diabetes (24), the frequency of OGTT-derived isolated postprandial hyperglycemia or its clinical course is well defined in sub-Saharan Africa, and will require further research. It is worth noting that of the two HbA_{1c} validation studies conducted among black populations in sub-Saharan Africa, Hird et al used both FPG and OGTT as diagnostic standards, with consistent results (AUROC 0.95 and 0.94, respectively)(26).

Not collecting OGTT data constitutes an important limitation of our study. In addition, although the overall sample size was large and the overall AUROC estimate was precise, the subgroups had fewer diabetes cases which resulted in our conducting descriptive rather than hypothesis-confirming analysis of subgroup validity. Another limitation is

For peer review only

that we did not assess the cause or type of anaemia, which would have had differential effects on HbA_{1c} as well as relevant implications for clinical practice.

Demographic models indicate that sub-Sahara will experience a substantial increase in diabetes prevalence in the coming years, which will require urgent strategies to scale up detection and treatment in order to increase access to care. As HbA_{1c} testing is less burdensome to patients than FPG and OGTT, it represents a useful option for expanding access to diabetes care. This will become particularly important as countries in sub-Saharan Africa allocate increasing resources to the health sector, and as operating costs for the HbA_{1c} test reduce over the next few years.

Figure legend

None

Funding

The work is supported primarily by the Wellcome Trust, United Kingdom [grant 098610Z/12/Z].

Competing interests

None declared.

Data Sharing

Study data are available on request from the Principle Investigators, Moffat Nyirenda (moffat.nyirenda@lshtm.ac.uk) and Amelia Crampin (mia.crampin@lshtm.ac.uk); the

For peer review only

participant consent allows for data sharing with research collaborators. The statistical analysis code is available here as supplemental material.

Author contributions

MJN, SJ and ACC conceived the study. SJ, ACC, CM, NK designed the protocols and implemented the study. LB designed the laboratory protocols and laboratory quality control, and implemented the laboratory work. JS and KB designed the data management protocols. EM designed the data cleaning protocols. SDR conducted data analysis. All authors wrote the manuscript.

Acknowledgments

We are grateful to the Karonga and Lilongwe communities, participants, and traditional authorities, for their engagement in this work. This study would also not have been possible without the encouragement and support of the Malawi Ministry of Health, particularly the Karonga and Lilongwe District Health Offices.

For peer review only

References

1. Ogurtsova K, da Rocha Fernandes JD, Huang Y, Linnenkamp U, Guariguata L, Cho NH, et al. IDF Diabetes Atlas: Global estimates for the prevalence of diabetes for 2015 and 2040. Diabetes Res Clin Pract. 2017 Jun;128:40–50.

2. Hilawe EH, Yatsuya H, Kawaguchi L, Aoyama A. Differences by sex in the prevalence of diabetes mellitus, impaired fasting glycaemia and impaired glucose tolerance in sub-Saharan Africa: a systematic review and meta-analysis. Bull World Health Organ. 2013 Sep 1;91(9):671–682D.

3. NCD Risk Factor Collaboration - Africa Working Group. Trends in obesity and diabetes across Africa from 1980 to 2014: an analysis of pooled population-based studies. Int J Epidemiol [Internet]. 2017 [cited 2017 Jul 19]; Available from: http://fdslive.oup.com/www.oup.com/pdf/production_in_progress.pdf

4. Mbanya JCN, Motala AA, Sobngwi E, Assah FK, Enoru ST. Diabetes in sub-Saharan Africa. The Lancet. 2010 Jun;375(9733):2254–66.

5. Burgess PI, Allain TJ, García-Fiñana M, Beare N a. V, Msukwa G, Harding SP. High prevalence in Malawi of sight-threatening retinopathy and visual impairment caused by diabetes: identification of population-specific targets for intervention. Diabet Med J Br Diabet Assoc. 2014 Dec;31(12):1643–50.

6. The International Expert Committee. International Expert Committee Report on the Role of the A1C Assay in the Diagnosis of Diabetes. Diabetes Care. 2009 Jul 1;32(7):1327–34.

For peer review only

7. American Diabetes Association. Diagnosis and Classification of Diabetes Mellitus. Diabetes Care. 2014 Jan 1;37(Supplement_1):S81–90.

8. World Health Organization. Use of glycated haemoglobin (HbA1c) in diagnosis of diabetes mellitus: abbreviated report of a WHO consultation [Internet]. 2011 [cited 2018 Feb 10]. Available from: http://www.who.int/diabetes/publications/report-hba1c_2011.pdf

9. Nakanga W, Crampin A, Nyirenda M. Personal View: Should haemoglobin A1C be used for diagnosis of diabetes mellitus in Malawi? Malawi Med J. 2016 Apr 6;28(1):28.

10. Son JI, Rhee SY, Woo J, Hwang JK, Chin SO, Chon S, et al. Hemoglobin A1c May Be an Inadequate Diagnostic Tool for Diabetes Mellitus in Anemic Subjects. Diabetes Metab J. 2013;37(5):343.

11. Kim C, Bullard KM, Herman WH, Beckles GL. Association Between Iron Deficiency and A1C Levels Among Adults Without Diabetes in the National Health and Nutrition Examination Survey, 1999-2006. Diabetes Care. 2010 Apr 1;33(4):780–5.

12. Little RR, Roberts WL. A review of variant hemoglobins interfering with hemoglobin A1c measurement. J Diabetes Sci Technol. 2009 May 1;3(3):446–51.

13. Slama L, Palella FJ, Abraham AG, Li X, Vigouroux C, Pialoux G, et al. Inaccuracy of haemoglobin A1c among HIV-infected men: effects of CD4 cell count,

For peer review only

antiretroviral therapies and haematological parameters. J Antimicrob Chemother. 2014 Dec 1;69(12):3360–7.

14. Glesby MJ, Hoover DR, Shi Q, Danoff A, Howard A, Tien P, et al. Glycated haemoglobin in diabetic women with and without HIV infection: data from the Women's Interagency HIV Study. Antivir Ther. 2010;15(4):571–7.

15. Herman WH. Do Race and Ethnicity Impact Hemoglobin A1c Independent of Glycemia? J Diabetes Sci Technol. 2009 Jul;3(4):656–60.

16. Cavagnolli G, Pimentel AL, Freitas PAC, Gross JL, Camargo JL. Effect of ethnicity on HbA1c levels in individuals without diabetes: Systematic review and meta-analysis. PLoS One. 2017 Feb 13;12(2):e0171315.

17. Crampin AC, Kayuni N, Amberbir A, Musicha C, Koole O, Tafatatha T, et al. Hypertension and diabetes in Africa: design and implementation of a large population-based study of burden and risk factors in rural and urban Malawi. Emerg Themes Epidemiol. 2016 Dec;13(3).

18. Msyamboza KP, Mvula CJ, Kathyola D. Prevalence and correlates of diabetes mellitus in Malawi: population-based national NCD STEPS survey. BMC Endocr Disord. 2014 May 12;14:41.

19. Crampin AC, Dube A, Mboma S, Price A, Chihana M, Jahn A, et al. Profile: The Karonga Health and Demographic Surveillance System. Int J Epidemiol. 2012 Jun 1;41(3):676–85.

For peer review only

20. World Health Organization, International Diabetes Federation. Definition and diagnosis of diabetes mellitus and intermediate hyperglycemia : report of a WHO/IDF consultation [Internet]. Geneva, Switzerland: World Health Organization; 2006. Available from: http://www.who.int/diabetes/publications/diagnosis_diabetes2006/en/

21. Youden WJ. Index for rating diagnostic tests. Cancer. 1950 Jan;3(1):32–5.

22. Mayega RW, Guwatudde D, Makumbi FE, Nakwagala FN, Peterson S, Tomson G, et al. Comparison of fasting plasma glucose and haemoglobin A1c point-of-care tests in screening for diabetes and abnormal glucose regulation in a rural low income setting. Diabetes Res Clin Pract. 2014 Apr;104(1):112–20.

23. Bertran EA, Berlie HD, Taylor A, Divine G, Jaber LA. Diagnostic performance of HbA_{1c} for diabetes in Arab vs. European populations: a systematic review and meta-analysis. Diabet Med. 2017 Feb;34(2):156–66.

24. Yan S, Liu S, Zhao Y, Zhang W, Sun X, Li J, et al. Diagnostic accuracy of HbA_{1c} in diabetes between Eastern and Western. Eur J Clin Invest. 2013 Jul;43(7):716–26.

25. Kodama S, Horikawa C, Fujihara K, Hirasawa R, Yachi Y, Yoshizawa S, et al. Use of high-normal levels of haemoglobin A1C and fasting plasma glucose for diabetes screening and for prediction: a meta-analysis. Diabetes Metab Res Rev. 2013 Nov;29(8):680–92.

26. Hird TR, Pirie FJ, Esterhuizen TM, O'Leary B, McCarthy MI, Young EH, et al. Burden of Diabetes and First Evidence for the Utility of HbA1c for Diagnosis and

For peer review only

Detection of Diabetes in Urban Black South Africans: The Durban Diabetes Study. Staiger H, editor. PLoS One. 2016 Aug 25;11(8):e0161966.

27. NCD Risk Factor Collaboration. Effects of diabetes definition on global surveillance of diabetes prevalence and diagnosis: a pooled analysis of 96 population-based studies with 331 288 participants. Lancet Diabetes Endocrinol. 2015 Aug;3(8):624–37.

For peer review only

Table 1. Demographic and clinical characteristics of participants in Karonga and Lilongwe, Malawi, 2013-2017.

	Total (n=3645) Median and IQR or n and %	Karonga (n=1613) Median and IQR, or n and %	Lilongwe (n=2032) Median and IQR, or n and %
Age, years	33 (25-44)	34 (26-47)	31 (24-41)
Female sex, %	63.0	58.3	67.2
Body mass index, kg/m^2	22.6 (20.5-26.0)	21.8 (20.0-23.8)	23.8 (21.2-28.0)
Normal BMI, %	61.1	81.9	57.8
Overweight BMI, %	20.4	13.8	26.1
Obese BMI, %	10.5	4.3	16.0
Systolic blood pressure, mm Hg	119 (111-129.5)	117.5 (110-127.5)	121 (112.5-131)
Diastolic blood pressure, mm Hg	72.5 (66.5-79.5)	72 (66-78)	73 (67-80.5)
Total cholesterol, mmol/l	3.8 (3.3-4.6)	3.8 (3.2-4.5)	3.9 (3.3-4.6)
Triglycerides, mmol/l	0.80 (0.58-1.14)	0.81 (0.58-1.12)	0.80 (0.59-1.18)

For peer review only

High density lipoprotein, mmol/l	1.12 (0.94-1.31)	1.09 (0.91-1.28)	1.15 (0.98-1.34)
Low density lipoprotein, mmol/l	2.53 (2.06-3.10)	2.44 (2.00-2.96)	2.62 (2.14-3.22)
Haemoglobin, g/dl	13.7 (12.6-14.9)	13.7 (12.6-15.0)	13.7 (12.6-14.9)
Normal haemoglobin, %	82.0	81.1	82.7
Anaemia, %	18.0	18.9	17.3
HIV positive, %	8.5	7.8	9.1
HbA_{1c} %	5.3 (4.9-5.7)	5.1 (4.7-5.5)	5.4 (5.1-5.8)
$HbA_{1c} \geq 6.5\%$ (48 mmol/mol), %	7.3	5.6	8.8
Fasting plasma glucose, mmol/l	4.7 (4.3-5.0)	4.7 (4.3-5.0)	4.7 (4.4-5.1)
FPG 6.1-7.0 mmol/L, %	1.8	1.7	1.9
FPG $\geq$ 7.0, %	1.7	1.3	2.1

FPG, Fasting plasma glucose; IRQ, Interquartile range

Aside counts, the figures are re-weighted by inverse probability of receiving HbA_{1c} testing

Table 2. Sensitivity, specificity, and positive and negative likelihood ratios for detecting type 2 diabetes at selected HbA_{1c} thresholds for participants in Karonga and Lilongwe, Malawi, 2013-2017.

HbA_{1c} threshold			Sensitivity (%)	Specificity (%)	Positive Likelihood Ratio	Negative Likelihood Ratio
%	mmol/ mol	SD				
6.0	42	+1.0	84.4	86.9	6.5	0.18
6.5*	48	+1.7	78.7	94.0	13.3	0.23
6.6†	49	+1.8	78.3	94.6	14.5	0.23
6.7	50	+2.0	76.5	96.0	19.0	0.25
7.4	57	+3.0	66.3	98.5	43.3	0.34
8.1	65	+4.0	54.8	99.5	117.4	0.45

SD, Standard Deviations from the mean HbA_{1c} score among diabetes-negative participants

* Diagnostic threshold recommended by American Diabetes Association, European Association for the Study of Diabetes, International Diabetes Federation and World Health Organization.

† Threshold identified by Youden's J statistic

For peer review only

Figures are re-weighted by the inverse probability of receiving HbA_{1c} testing

For peer review only

Table 3. Overall and stratum-specific area under the receiver operating characteristic (AUROC) curves for detection of type 2 diabetes with HbA_{1c} among adults in Malawi, 2013-2017.

	AUROC (95% CI)	Sensitivity (%)*	Specificity (%)*	Positive Likelihood Ratio*	Negative Likelihood Ratio*
Overall	0.92 (0.90-0.94)	78.7	94.0	13.3	0.23
Site					
Karonga	0.88 (0.94-0.92)	70.2	95.4	15.2	0.31
Lilongwe	0.94 (0.92-0.96)	83.3	92.9	11.7	0.18
Sex					
Female	0.93 (0.91-0.95)	79.1	95.2	16.5	0.22
Male	0.89 (0.85-0.93)	77.6	92.1	9.9	0.24
Body mass index					
Normal	0.87 (0.83-0.92)	70.1	95.1	14.5	0.31
Overweight	0.93 (0.89-0.96)	84.5	91.4	9.9	0.17
Obese	0.92 (0.89-0.95)	80.4	90.6	8.6	0.22

For peer review only

Haemoglobin					
Normal	0.93 (0.91-0.95)	81.4	93.5	12.6	0.20
Anaemia	0.86 (0.79-0.93)	61.5	96.4	17.4	0.40

* At HbA1c $\geq$ 6.5%

Figures are re-weighted by the inverse probability of receiving HbA_{1c} testing

For peer review only

Table 4. Clinical characteristics of participants identified as having diabetes by fasting blood glucose, and for true- and false-positive participants by $HbA_{1c} \geq 6.5\%$ in Karonga and Lilongwe, Malawi, 2013-2017.

	Type 2 diabetes positive by FPG ≥7.0 mmol/L (IQR)	Type 2 diabetes true positive by $HbA_{1c} \geq 6.5\%$* (IQR)	Type 2 diabetes false positive by $HbA_{1c} \geq 6.5\%$*† (IQR)
Age, years	50 (41-60)	51 (43-61)	35 (23-51)
Female sex, %	65.6	66.3	51.0
Body mass index, kg/m^2	27.9 (24.2-32.5)	28.2 (24.8-32.5)	24.8 (20.9-28.3)
Systolic blood pressure, mm Hg	133.5 (121.5-153)	135 (123-156)	125 (115-136)
Diastolic blood pressure, mm Hg	81.5 (74.5-89.5)	81.5 (75.0-90.5)	74.5 (68.5-82.5)
Total cholesterol, mmol/L	4.9 (4.1-5.7)	5.0 (4.3-5.8)	4.2 (3.5-5.2)
Triglycerides, mmol/L	1.7 (1.2-2.3)	1.8 (1.2-2.5)	0.9 (0.6-1.5)
High density lipoprotein, mmol/L	1.04 (0.89-1.22)	1.03 (0.88-1.20)	1.15 (0.97-1.29)

For peer review only

Low density lipoprotein, mmol/L	3.38 (2.69-4.09)	3.52 (2.89-4.22)	2.82 (2.36-3.56)
Haemoglobin, g/dl	13.9 (12.9-14.9)	13.9 (12.9-15.0)	14.2 (13.1-15.1)
HbA_{1c}, %	8.4 (6.9-11.3)	9.6 (7.7-11.8)	6.9 (6.6-7.4)
Fasting plasma glucose, mmol/L	9.5 (7.7-14.2)	10.7 (8.3-15.4)	4.8 (4.5-5.1)

FPG, Fasting Plasma Glucose; IQR, Interquartile range

* Diagnostic threshold recommended by American Diabetes Association, European Association for the Study of Diabetes, World Health Organization, and International Diabetes Federation.

Aside counts, figures are weighted by the inverse probability of receiving HbA_{1c} testing

For peer review only

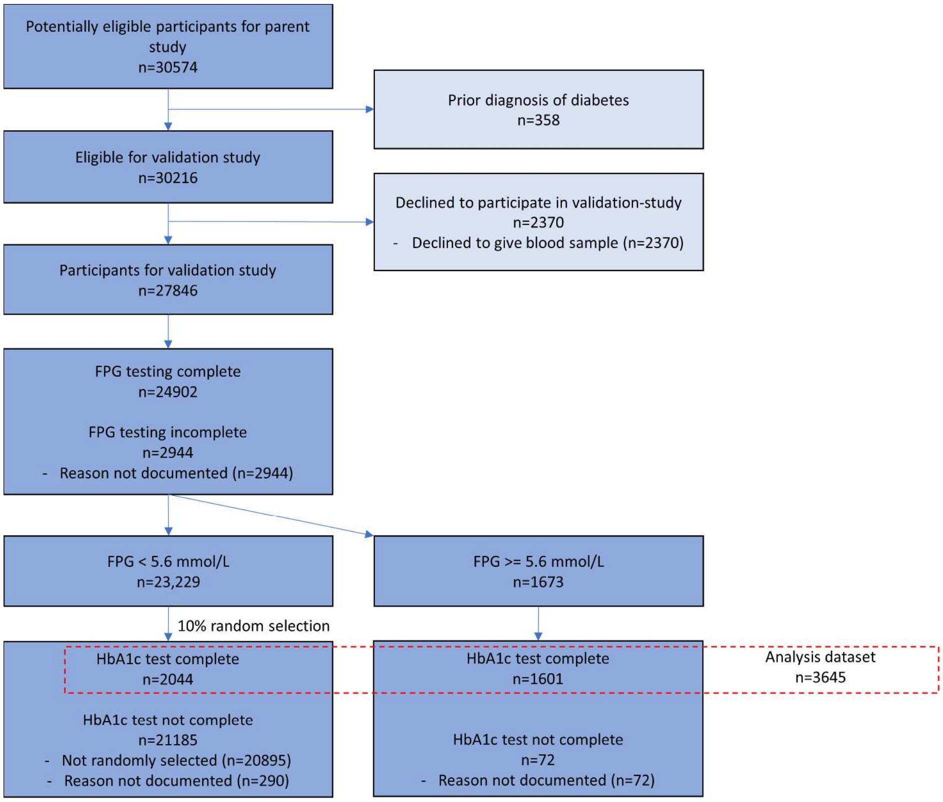

STARD diagram

250x230mm (300 x 300 DPI)

```
*******************
*** Hb1Ac AUROC ***
*******************
      use "30Aug17.dta", clear
      keep gluc_units hba1_units ///
            BMI systolic diastolic chol_units tg_units hdl ldl hgb ///
            sex site stid age
      qui compress
      sum

****************
*** NEW VARS ***
****************************************************************
      gen diabetes_fpg=(gluc_units>=7.0)
      label variable diabetes_fpg "Newly diabetes positive (fasting plasma glucose>=7)"
      label define yesno 0 No 1 Yes
      label values diabetes_fpg yesno

      recode gluc_units (min/6.09=0)(6.10/6.99=1)(7.0/max=0), gen(prediabetes)
      label variable prediabetes "Pre-diabetes (fasting glucose of 6.1-6.9)"
      label values prediabetes yesno

      recode gluc_units (min/5.59=0)(5.6/max=1), gen(gluc56)
      label variable gluc56 "Fasting glucose >=5.6 mmol/L"
      label values gluc56 yesno

      recode hba1 (min/6.49=0)(6.50/max=1), gen(hba65)
      label variable hba65 "Diabetes screen positive (HbA1c >= 6.5%)"
      label values hba65 yesno

      recode hba1 (min/6.79=0)(6.80/max=1), gen(hba68)
      label variable hba68 "Diabetes screen pos (HbA1c >=6.8%)"
      label values hba68 yesno

      gen hbaint = round(hba1_units,1)
      label variable hbaint "HbA1c score, rounded to integer"

      gen hbadec = round(hba1_units,0.1)
```

```
label variable hbaint "HbA1c score, rounded to tenths"

encode sex, gen(gender)
label define gender 1 Female 2 Male, modify
label values gender gender
label variable gender "Sex"
drop sex
rename gender sex

gen tempsite = site=="L"
label define site 0 Karonga 1 Lilongwe, modify
label values tempsite site
drop site
rename tempsite site
label variable site "Research site"

gen hbatest = !mi(hba1)
gen pw =!mi(hba1)
recode pw (1=10) if gluc_units<5.6
label variable pw "Probability sampling weight for HbA1c testing"

sort stid
gen pid = _n
label variable pid "Unique identifier"
expand=pw
summarize hba1_unit if diabetes_fpg==0
gen meanhba = r(mean)
gen sdhba = r(sd)
gen zscore = (hba1_units - meanhba) / sdhba
label variable zscore "HbA1c z-score"
notes zscore: Using only HbA1c scores from diabetes-negative pts and re-weighted for the sub-sampling
duplicates drop pid, force

gen zdec = round(zscore, 0.1)
label variable zdec "Normal HbA1c z-score, rounded to 0.1"

recode BMI (min/24.9999=0)(25/29.999=1)(30/max=2), gen(bmi_3c)
label define bmi 0 Normal 1 Overweight 2 Obese
```

For peer review only

```
      label values bmi_3c bmi
      label variable bmi_3c "BMI category"

      gen anemia=0
      replace anemia=1 if sex==2 & hgb<13.5
      replace anemia=1 if sex==1 & hgb<12.0
      label values anemia yesno

      quiet compress

      tempfile full
      save `full'
*** END NEW VARS ***********************************************

*** FLOW CHART ***
      use `full', clear
      count
      drop if mi(gluc_units)
      drop if mi(hba1)
      tab gluc56, sum(hba1)

**************************
*** DESCRIPTIVE CHECKS ***
**************************
      sum age sex site hba1 gluc_units diabetes_fpg pw    /* Most of these are skewed */
      logistic diabetes_fpg hba1_units [pw=pw]
      prop diabetes_fpg
      prop diabetes_fpg [pw=pw]


****************************************
*** TABLE 1: Patient characteristics ***
****************************************
      tabstat age sex BMI syst dias chol_units tg_units hdl_units ldl_units hgb hba1_units gluc_units[aw=pw], 
stat(n p50 p25 p75) by(site) f(%9.2f) col(s)
      foreach var of varlist sex bmi_3c anemia hba65 prediabetes diabetes_fpg {
            prop `var' [pw=pw]
            prop `var' [pw=pw], over(site)
```

For peer review only

```
            }
      replace prediabetes=. if gluc_units>=7.0

******************************
*** TABLE 2: Validity stats ***
******************************
      * Overall dataset for HbA1c vs diabetes
      foreach x of varlist diabetes_fpg prediabetes {
            somersd `x' hba1 [pw=pw], trans(c)
            roctab `x' hba1, binomial sum
            senspec `x' hbadec [pw=pw], sensitivity(sens) specificity(spec) ntpos(tp) nfpos(fp) ntneg(tn) 
nfneg(fn)
            for var sens spec tp fp tn fn: replace X = 100*X
            gen ppv = 100*tp / (tp+fp)
            gen npv = 100*tn / (tn+fn)
            gen lrpos = sens / (100-spec)
            gen lrneg = (100-sens) / spec
            gen jtemp = sens + spec - 100
            egen youdenj = max(jtemp)
            replace youdenj=. if youdenj!=jtemp
            table zdec, c(mean sens mean spec mean youdenj mean lrpos mean lrneg) format(%9.2f)
            drop sens spec tp fp tn fn ppv npv lrpos lrneg jtemp youdenj
      }

***************
*** TABLE 3 ***
***************
      * Stratum-specific HbA1c vs diabetes
      foreach var of varlist site sex bmi_3c anemia {
            levelsof `var', local(levels)
            foreach l of local levels {
                  tab `var' if `var'==`l'
                  somersd diabetes_fpg hba1 [pw=pw] if `var'==`l', trans(c)
                  senspec diabetes_fpg hbadec [pw=pw] if `var'==`l', sensitivity(sens) specificity(spec)
                  for var sens spec: replace X = 100*X
                  gen lrpos = sens / (100-spec)
                  gen lrneg = (100-sens) / spec
```

For peer review only

```
                table zdec if zdec>1.66 & zdec<1.71 & `var'==`l', c(mean sens mean spec mean lrpos mean lrneg) format(%9.2f)
                drop sens spec lrpos lrneg
                }
        }

*******************************
*** Table 4: Diabetes chars ***
*******************************
* Present clinical charteristics of diabetes+ by fasting glucose criteria
      tabstat age sex BMI syst dias chol_units tg_units hdl_units ldl_units hgb hba1_units gluc_units  if diabetes_fpg [aw=pw], stat(p50 p25 p75) f(%9.2f) col(s)
      prop sex [pw=pw] if diabetes_fpg

* Present clinical characteristics among HbA1c+, by true status
      tab hba65 diabetes_fpg
      tabstat age sex BMI syst dias chol_units tg_units hdl_units ldl_units hgb hba1_units gluc_units  if hba65 [aw=pw], by(diabetes_fpg) stat(p50 p25 p75) f(%9.2f) col(s)
      prop sex if hba65 [pw=pw], over(diabetes_fpg)
```

For peer review only

Section & Topic	No	Item	Reported on page #
**TITLE OR ABSTRACT**			
	**1**	Identification as a study of diagnostic accuracy using at least one measure of accuracy (such as sensitivity, specificity, predictive values, or AUC)	1
**ABSTRACT**			
	**2**	Structured summary of study design, methods, results, and conclusions (for specific guidance, see STARD for Abstracts)	3
**INTRODUCTION**			
	**3**	Scientific and clinical background, including the intended use and clinical role of the index test	5
	**4**	Study objectives and hypotheses	6
**METHODS**			
Study design	**5**	Whether data collection was planned before the index test and reference standard were performed (prospective study) or after (retrospective study)	6
Participants	**6**	Eligibility criteria	6
	**7**	On what basis potentially eligible participants were identified (such as symptoms, results from previous tests, inclusion in registry)	6
	**8**	Where and when potentially eligible participants were identified (setting, location and dates)	6
	**9**	Whether participants formed a consecutive, random or convenience series	6
Test methods	**10a**	Index test, in sufficient detail to allow replication	7
	**10b**	Reference standard, in sufficient detail to allow replication	7
	**11**	Rationale for choosing the reference standard (if alternatives exist)	15
	**12a**	Definition of and rationale for test positivity cut-offs or result categories of the index test, distinguishing pre-specified from exploratory	8
	**12b**	Definition of and rationale for test positivity cut-offs or result categories of the reference standard, distinguishing pre-specified from exploratory	7
	**13a**	Whether clinical information and reference standard results were available to the performers/readers of the index test	7
	**13b**	Whether clinical information and index test results were available to the assessors of the reference standard	7
Analysis	**14**	Methods for estimating or comparing measures of diagnostic accuracy	8
	**15**	How indeterminate index test or reference standard results were handled	NA
	**16**	How missing data on the index test and reference standard were handled	NA
	**17**	Any analyses of variability in diagnostic accuracy, distinguishing pre-specified from exploratory	8
	**18**	Intended sample size and how it was determined	6
**RESULTS**			
Participants	**19**	Flow of participants, using a diagram	Figure 1
	**20**	Baseline demographic and clinical characteristics of participants	11
	**21a**	Distribution of severity of disease in those with the target condition	NA
	**21b**	Distribution of alternative diagnoses in those without the target condition	NA
	**22**	Time interval and any clinical interventions between index test and reference standard	6
Test results	**23**	Cross tabulation of the index test results (or their distribution) by the results of the reference standard	10-11
	**24**	Estimates of diagnostic accuracy and their precision (such as 95% confidence intervals)	11
	**25**	Any adverse events from performing the index test or the reference standard	11
**DISCUSSION**			
	**26**	Study limitations, including sources of potential bias, statistical uncertainty, and generalisability	15
	**27**	Implications for practice, including the intended use and clinical role of the index test	12-13
**OTHER INFORMATION**			
	**28**	Registration number and name of registry	NA
	**29**	Where the full study protocol can be accessed	NA
	**30**	Sources of funding and other support; role of funders	16

For peer review only - http://bmjopen.bmj.com/site/about/guidelines.xhtml

CC BY SA

BMJ Open

Glycated hemoglobin A1c (HbA1c) for detection of diabetes mellitus and impaired fasting glucose in Malawi: a diagnostic accuracy study

Journal:	*BMJ Open*
Manuscript ID	bmjopen-2017-020972.R2
Article Type:	Research
Date Submitted by the Author:	26-Mar-2018

Complete List of Authors:	Rathod, Sujit; London School of Hygiene and Tropical Medicine, Department of Population Health<br>Crampin, Amelia; London School of Hygiene and Tropical Medicine<br>Musicha, Crispin; Malawi Epidemiology and Intervention Research Unit<br>Kayuni, Ndoliwe; Malawi Epidemiology and Intervention Research Unit<br>Banda, Louis; Malawi Epidemiology and Intervention Research Unit<br>Saul, Jacqueline; London School of Hygiene and Tropical Medicine<br>McLean, Estelle; London School of Hygiene and Tropical Medicine; Malawi Epidemiology and Intervention Research Unit<br>Branson, Keith; London School of Hygiene and Tropical Medicine<br>Jaffar, Shabbar; Liverpool School of Tropical Medicine<br>Nyirenda, Moffat; London School of Hygiene and Tropical Medicine

**Primary Subject Heading**:	Diabetes and endocrinology
Secondary Subject Heading:	Diagnostics
Keywords:	HbA1c, Type 2 diabetes, Malawi, validity, receiver operating characteristic

SCHOLARONE™ Manuscripts

Glycated hemoglobin A_{1c} (HbA_{1c}) for detection of diabetes mellitus and impaired fasting glucose in Malawi: a diagnostic accuracy study

Sujit D Rathod*,[1] Amelia C Crampin,[1] Crispin Musicha,[2] Ndoliwe Kayuni,[2] Louis Banda,[2] Jacqueline Saul,[1] Estelle McLean,[1,2] Keith Branson,[1] Shabbar Jaffar,[3] Moffat J Nyirenda,[1]

[1] London School of Hygiene & Tropical Medicine, London, United Kingdom

[2] Malawi Epidemiology and Intervention Research Unit, Chilumba, Malawi

[3] Liverpool School of Tropical Medicine, Liverpool, United Kingdom

*Corresponding author

Department of Population Health

London School of Hygiene & Tropical Medicine

Keppel Street

London WC1E7HT

For peer review only

United Kingdom

P: +44 020 7299 4683

F: +44 (0)20 7436 4230

sujit.rathod@lshtm.ac.uk

For peer review only

Abstract

Objectives: To examine the accuracy of glycated hemoglobin A_{1c} (HbA_{1c}) in detecting type 2 diabetes and impaired fasting glucose among adults living in Malawi.

Design: A diagnostic validation study of HbA_{1c}. Fasting plasma glucose (FPG) ≥7.0 mmol/L was the reference standard for Type 2 diabetes, and FPG between 6.1 and 6.9 mmol/L as impaired fasting glucose.

Participants: 3645 adults (of whom 63% female) recruited from two demographic surveillance study sites in urban and rural Malawi. This analysis excluded those who had a previous diagnosis of diabetes or had history of taking diabetes medication.

Results: HbA_{1c} demonstrated excellent validity to detect FPG-defined diabetes, with an area under the receiver operating characteristic (AUROC) curve of 0.92 (95% CI 0.90-0.94). At HbA_{1c}≥6.5% (48 mmol/mol), sensitivity was 78.7% and specificity was 94.0%. Subgroup AUROCs ranged from 0.86 for participants with anaemia to 0.94 for participants in urban Malawi. There were clinical and metabolic differences between participants who were true diabetes vs. false positives when HbA_{1c}≥6.5% (48 mmol/mol).

Conclusions: The findings from this study provide justification to use HbA_{1c} to detect type 2 diabetes. As HbA_{1c} testing is substantially less burdensome to patients than either fasting plasma glucose testing or oral glucose tolerance testing, it represents a useful option for expanding access to diabetes care in sub-Saharan Africa.

For peer review only

Strengths and limitations of this study

- Only two studies have assessed the validity of HbA_{1c} to detect type 2 diabetes in African-origin populations; this is the first such study from Malawi.
- We used fasting plasma glucose as the reference standard, which is commonly used in clinical practice and for validation studies.
- The results of this study may not be generalizable to other populations in sub-Saharan Africa, or those with anaemia.

For peer review only

Over 400 million people live with type 2 diabetes worldwide, with more than 75% of these in low- and middle-income countries (1). While the prevalence of diabetes in sub-Saharan African is lower than in other global regions, around 6-9% (2,3), demographic models project that this region will experience the fastest growth rate in cases over the next 20 years (1). Approximately 50-66% of people with diabetes are undiagnosed (1,4), and complications are common even after diagnosis (5). As barriers to diabetes care become surmounted in this region (4), focus must turn to improving the accessibility of diagnostics.

The diagnosis of diabetes has traditionally been based upon the detection of elevated plasma glucose levels, either after fasting or two hours after an oral glucose tolerance test, or, in symptomatic individuals, after a random blood glucose check (6). While these tests are available in sub-Saharan Africa, access to services are limited, travel times and clinic waiting times are high, and integrity of the samples and quality of the measuring tools are in most cases uncertain. More reliable approaches to circumvent some of these challenges are required. Recently, the American Diabetes Association (ADA) and the World Health Organization (WHO) have recommended using glycated hemoglobin A_{1c} (HbA_{1c}) in blood to diagnose diabetes mellitus (7,8). HbA_{1c} testing provides significant practical advantages over glucose testing as it does not fluctuate appreciably and thus can be performed at any time of the day. Further, HbA_{1c} testing does not require any special pre-test preparations, such as overnight fasting or glucose loading.

For peer review only

There are acknowledged limitations when attempting to measure HbA_{1c} levels which may be relevant for diagnostics in sub-Saharan Africa (6,8,9). It is unclear whether one can use HbA_{1c} to diagnose diabetes for people who have malaria (6), haemolytic anaemia (10,11), sickle cell anaemia (12), HIV infection (13,14) or who are of African descent (15,16). Given uncertainty around the validity of HbA_{1c} to diagnose type 2 diabetes and its precursor, impaired fasting glucose, in sub-Saharan Africa, we conducted a diagnostic accuracy study in Malawi, and have reported results here.

Design and Methods

Study setting

This report uses data collected as part of a Malawi Epidemiology and Intervention Research Unit (MEIRU) survey, which aimed to understand the burden and risk factors of non-communicable diseases in Malawi (17), where the national prevalence of diabetes is estimated to be 5.6% (18). Community-based cross-sectional surveys were conducted in Karonga District (May 2013-April 2016) (19) and Lilongwe City (June 2013-April 2017) (17). Karonga is a rural, low-altitude, malaria-endemic district in northern Malawi, and Lilongwe is an urban, high altitude city in central Malawi with lower malaria prevalence.

Recruitment and data collection

Detailed study procedures have been previously reported (17). All adults aged 18 years and above who were usually resident in either of the study sites were eligible to

For peer review only

participate in the parent study. All households were approached consecutively, and all residents aged ≥18 years were recruited. Recruits provided written informed consent for each separate study component (i.e. standardized interview, physical measurements and blood specimen collection) and could opt out of any component. Venipuncture was conducted after a minimum 8-hour fasting period and whole blood samples were collected in sodium fluoride tubes. Tubes were stored on ice in an insulated cool box and delivered to the laboratory for processing (mean delivery time of 2.6 hours after blood collection), and glucose analysis was completed within 1 hour after processing. Quantitative determination of fasting plasma glucose (hexokinase method) and HbA_{1c} was performed using the Beckman Coulter Chemistry Analyzer AU480 system according to the manufacturer's guidelines.

FPG testing was conducted on all participant samples. HbA_{1c} testing was conducted on all participants samples for which FPG results were greater than 5.6 mmol/L and in a 10% random sample of those with FPG lower than 5.6 mmol/L. The lab technician who conducted the HbA_{1c} test was blind to the participants' clinical characteristics and FPG result. Nearly all (87%) of HbA_{1c} tests were completed within 24 hours of FPG test. Cut-points recommended by WHO were used to define fasting plasma glucose (FPG) as normal (<6.1 mmol/L), impaired fasting glucose (6.1 to 6.9 mmol/L) or diabetes (≥7.0 mmol/L) (20). Categories of body mass index (BMI) for underweight/normal (BMI<25kg/m^2), overweight (25≤BMI<30) and obese (BMI≥30), and categories for anaemia (haemoglobin<12.0g/dL for women and <13.5 for men) were used.

Statistical analyses

Participants who reported a past diagnosis of diabetes or history of taking diabetes medication were excluded from this analysis. First, demographic and clinical variables (i.e. age, sex, location, BMI, blood pressure, plasma glucose and lipid profile, and HIV serostatus) were described separately for the Karonga and Lilongwe samples. Medians and interquartile ranges were reported for the continuous measures, as most of these measures were skewed, and proportions for categorical measures. Second, distribution of HbA_{1c} level was compared by FPG result (<7.0 vs ≥7.0 mmol/L) with logistic regression. Third, the validity of HbA_{1c} for diagnosing diabetes, using FPG (>7.0 mmol/L) as reference standard, was assessed by using Somers' D statistic with Harrell's C transformation to estimate the area under the receiver operating characteristics (AUROC) curve and 95% confidence interval. Validity statistics (i.e. sensitivity, specificity, and positive and negative likelihood ratios) were reported for standardized HbA_{1c} scores which correspond to 1.0, 2.0, 3.0 and 4.0 standard deviations above the sample's mean HbA_{1c} score, at an 'optimal' value identified by the Youden's J statistic (where the sum of the sensitivity and specificity reaches its maximum) (21), and at 6.5% (48 mmol/mol), which is the value recommended by several diabetes associations. The standardized values were generated using the distribution of HbA_{1c} scores from the diabetes-negative participants with HbA_{1c} test results (i.e. all participants with fasting plasma glucose ≥5.6 mmol/L and a 10% sub-sample of participants with plasma glucose <5.6) such that the sub-sampled participants were upweighted 10-fold before generating the distribution. Fourth, to consider the validity of HbA_{1c} to detect diabetes across subgroups, the ROC

For peer review only

analysis was stratified by site, sex, BMI, and hemoglobin level. Within each stratum the sensitivity, specificity, positive and negative likelihood ratio when using HbA_{1c}≥6.5% (48 mmol/mol) as a cut off score were reported. Fifth, the AUROC and 95% CI for detecting impaired fasting glucose (FPG ≥ 6.1 mmol/L) with HbA_{1c} was estimated among those who did not have diabetes (FPG <7.0 mmol/L). Finally, the clinical characteristics were described for the subsets of participants who had FPG-defined diabetes, and for true- and false-positive participants with HbA_{1c}≥6.5% (48 mmol/mol). Again, medians and interquartile ranges were reported for continuous measures and proportions for categorical measures. The analysis was conducted using Stata SE 14.2 (StataCorp, College Station, Texas, USA)(See Stata code in supplemental material) with complete-case analysis. Aside count figures, the statistical results were weighted by the inverse probability of receiving the HbA_{1c} test.

Ethical approval

The study protocol was reviewed and approved by the Malawi National Health Sciences Research Committee, and London School of Hygiene and Tropical Medicine (UK) Ethics Committee. Participants identified as having diabetes were referred to chronic care clinics established in conjunction with the Malawi Ministry of Health, where they initiated management per national treatment guidelines.

Patient and public involvement

For peer review only

The Karonga and Lilongwe population platforms' research priorities have reflected the Malawi National Health Research Agenda, and operate through a longstanding research partnership with the Malawi National Ministry of Health and Malawi College of Medicine. The design and aims of these platforms were developed in a stakeholders' meeting in Lilongwe, the capital city of Malawi, attended by policy makers, policy implementers and researchers. The designs of the platforms were influenced by staff working closely with the communities and engaging through community meetings. Prior to launching new sub-studies, the research team conducts community sensitization events with dancing, dramas and question and answer sessions. In Karonga, village elders and other responsible community members were responsible for enumerating households, and for reporting household vital events and household migrations. In Lilongwe, block leaders were involved in sensitization and communication with the community prior to enumeration by research staff. Preliminary results from this study have been disseminated at a conference held at the University of Malawi College of Medicine, and presentations for lay audiences, such as for the participating communities, are being prepared.

Results

As shown in the STARD flow diagram (Figure 1), 30,574 adults consented to the interview and clinical examination in the parent study, of whom 27,846 also provided consent for blood specimen collection, as was required for this sub-study. No participant experienced a severe adverse event due to the specimen collection. This analysis includes the 3645 participants (1613 in Karonga and 2032 in Lilongwe) who had both FPG and

For peer review only

HbA_{1c} levels measured, and who did not report prior diagnosis of diabetes by a medical professional.

Demographic and Clinical Characteristics

The demographic and clinical characteristics of analysis participants are reported in Table 1.

For the 3645 participants, the median age was 33 years (IQR 24-44) and 63% were female. The median HbA_{1c} level was 5.3% (IQR 4.9-5.7) and 7.3% had HbA_{1c} level of 6.5% (48 mmol/mol) or higher. The median FPG level was 4.7 mmol/L (IQR 4.3-5.0); 1.8% of participants had a FPG level consistent with impaired fasting glucose and another 1.7% with diabetes.

Diagnostic validity of HbA_{1c}

For every percentage unit increase in HbA_{1c} score, there was an almost 3-fold increase in the odds of having FPG-defined diabetes (OR 2.80, 95% CI 2.21-3.53, R^2 0.371, Wald $X^2(1)$ 75.5, $P<0.001$). Further, HbA_{1c} demonstrated excellent validity to detect for FPG-defined diabetes, with an AUROC of 0.92 (95% CI 0.90-0.94). Validity statistics are presented for a range of HbA_{1c} cut points in Table 2.

An HbA_{1c} value of 6.0% corresponded to 1.0 standard deviation above the mean in the diabetes-negative sample's distribution of HbA_{1c} scores; at this cut point 84.4% of people with diabetes would test positive (sensitivity) and 86.9% of people without diabetes

For peer review only

would test negative (specificity). At the commonly-recommended HbA_{1c} threshold of 6.5% (48 mmol/mol), sensitivity was 78.7% and specificity was 94.0%. Youden's J was at an HbA_{1c} value of 6.6%, where sensitivity was 78.3% and specificity was 94.6%. Stratum-specific AUROCs (Table 3), ranged from a minimum of 0.86 for participants with anaemia to a maximum of 0.94 for participants in Lilongwe. With $HbA_{1c}\geq6.5\%$ (48 mmol/mol), participants with anaemia had 61.5% sensitivity, 96.4% specificity and a positive likelihood ratio of 17.4, and the corresponding figures for participants in Lilongwe were 70.2%, 95.4%, and 15.2.

Among participants who did not have FPG-defined diabetes, the HbA_{1c} score had average validity to detect impaired fasting glucose (AUROC 0.70, 95% CI 0.67-0.73).

Clinical characteristics of participants meeting diagnostic criteria for diabetes

The clinical characteristics of participants who had FPG-defined diabetes, and for participants who had true positive and false positives results are presented in Table 4.

The participants who had FPG-defined diabetes (FPG $\geq$7.0 mmol/L) had a median age of 50 years (IQR 41-60) and 65.5% were female. The median BMI was 27.9 kg/m^2 (IQR 24.2-32.5) and the median systolic blood pressure 133.5 mm Hg (IQR 121.5-153). The characteristics of true positive participants (HbA_{1c} $\geq$6.5% (48 mmol/mol) and FPG$\geq$7.0 mmol/L) and the false positive participants (HbA_{1c} $\geq$6.5% (48 mmol/mol) and FPG< 7.0 mmol/L) are also described in Table 4..

For peer review only

Discussion

In a large, multi-site sample of adults in Malawi, we found that HbA_{1c} is highly predictive of FPG-defined type 2 diabetes, a relationship which was consistent across several sub-groups. HbA_{1c} was less predictive for impaired fasting glucose. Using a cut off value of HbA_{1c} 6.5% (48 mmol/mol) to detect diabetes, there were demographic and clinical differences between true- and false-positive participants.

This analysis is one of the first to compare HbA_{1c} as a stand-alone test to detect type 2 diabetes in a black African population in sub-Saharan Africa, and the first from Malawi. In Uganda, Mayega et al found that HbA_{1c} had moderate validity (AUROC 0.75) for detecting FPG-defined diabetes (22), which was lower than found by Hird et al in South Africa (AUROC 0.95). These findings from sub-Saharan Africa complement meta-analyses of studies in East Asia, Middle East and Europe which show that HbA_{1c} is a valid test for detecting diabetes (23–25) across different ethnic groups.

There were several notable demographic and clinical differences between participants who were true vs false positives for FPG-defined diabetes at $HbA_{1c} \geq 6.5\%$ (48 mmol/mol). Compared to participants who had $HbA_{1c} \geq 6.5\%$ (48 mmol/mol) and FPG<7.0 mmol/L, those who had both FPG≥7.0 and $HbA_{1c} \geq 6.5\%$ (48 mmol/mol) were more likely to be female, to be older, and to have higher BMI and blood pressure. These findings are consistent with data from the Uganda where the AUROC for all participants was 0.75, but was significantly higher at 0.90 for overweight participants and 0.98 for obese participants. This suggests that HbA_{1c} is particularly useful in in detecting diabetes

in individuals with insulin resistance or metabolic syndrome (e.g. constellation of high BMI, hypertension, dyslipidemia). In contrast, the false positive group (HbA_{1c}≥6.5% (48 mmol/mol) and FPG<7.0 mmol/L) lacked features of metabolic syndrome. The false positive group is likely to include individuals with isolated postprandial hyperglycemia, rather than fasting hyperglycemia, (perhaps from pancreatic deficiency rather than insulin resistance) which would require oral glucose tolerance testing (OGTT) to demonstrate. Notably, HbA_{1c} has been shown to strongly predict OGTT-defined diabetes in South Africa, where Hird et al estimated the AUROC at 0.94 (26). The clinical and demographic differences between true- and false- positive participants observed here, though interesting, remain descriptive and will require confirmation in larger studies.

We found that HbA_{1c} had poor predictive ability for detecting impaired fasting glucose. This is consistent with other studies in Africa and evidence from a recent meta-analysis by Kodama et al (22,25,26). Accordingly, evidence strongly suggests that HbA_{1c} may not be appropriate for detecting impaired fasting glucose in this population.

Stratum-specific AUROC analysis indicated largely consistent values across strata (i.e. by sex, site, and BMI), which is evidence of suitability of HbA_{1c} to detect diabetes across a range of groups. However, AUROC value were lowest for those with anaemia – which appears to be driven by a drop in sensitivity. Different forms of anaemia (e.g. chronic or acute) affect the integrity and quantity of HbA1c-carrying red blood cells, resulting in HbA_{1c} having diminished utility as a stand-alone diagnostic tool among individuals with anaemia (11). For example, Son et al also found that the AUROC for HbA_{1c} to detect

For peer review only

OGTT-defined diabetes in South Korea was lower for adults with anaemia (0.86 vs 0.88) (10). Future research will be required to determine how to control anaemia in sub-Saharan Africa so that HbA_{1c} testing maintains its validity.

We used FPG rather than OGTT as the reference standard for diabetes in this analysis. A 2013 meta-analysis of 13 cohort studies found that FPG is highly correlated to present and to future diabetes (25), though none of the studies identified were from sub-Saharan Africa. A 2015 meta-analysis of diabetes measures found 27 validation studies of HbA_{1c} using FPG-defined diabetes as the diagnostic standard, compared to 9 using OGTT (27). FPG is typically preferred for validation studies due to its practical advantages, though it is unclear what proportion of individuals with diabetes is missed when FPG testing is not paired with OGTT. While FPG is highly correlated to present and to future diabetes (24), the frequency of OGTT-derived isolated postprandial hyperglycemia or its clinical course is not well defined in sub-Saharan Africa, and will require further research. It is worth noting that of the two HbA_{1c} validation studies conducted among black populations in sub-Saharan Africa, Hird et al used both FPG and OGTT as diagnostic standards, with consistent results (AUROC 0.95 and 0.94, respectively)(26).

Not collecting OGTT data constitutes an important limitation of our study. In addition, although the overall sample size was large and the overall AUROC estimate was precise, the subgroups had fewer diabetes cases which resulted in our conducting descriptive rather than hypothesis-confirming analysis of subgroup validity. Another limitation is that we did not assess the cause or type of anaemia, which would have had differential

For peer review only

effects on HbA_{1c} as well as relevant implications for clinical practice. A final limitation concerns the decay in glucose levels which occur when blood is stored in fluoride tubes. The reduction in variation in glucose levels was independent of OGTT result, and so would have resulted our underestimating the AUROCs for diabetes and for impaired fasting glucose.

Demographic models indicate that sub-Sahara will experience a substantial increase in diabetes prevalence in the coming years, which will require urgent strategies to scale up detection and treatment in order to increase access to care. As HbA_{1c} testing is less burdensome to patients than FPG and OGTT, it represents a useful option for expanding access to diabetes care. This will become particularly important as countries in sub-Saharan Africa allocate increasing resources to the health sector, and as operating costs for the HbA_{1c} test reduce over the next few years.

For peer review only

Figure legend

None

Funding

The work is supported primarily by the Wellcome Trust, United Kingdom [grant 098610Z/12/Z].

Competing interests

None declared.

Data Sharing

Study data are available on request from the Principle Investigators, Moffat Nyirenda (moffat.nyirenda@lshtm.ac.uk) and Amelia Crampin (mia.crampin@lshtm.ac.uk); the participant consent allows for data sharing with research collaborators. The statistical analysis code is available here as supplemental material.

Author contributions

MJN, SJ and ACC conceived the study. SJ, ACC, CM, NK designed the protocols and implemented the study. LB designed the laboratory protocols and laboratory quality control, and implemented the laboratory work. JS and KB designed the data management protocols. EM designed the data cleaning protocols. SDR conducted data analysis. All authors wrote the manuscript.

Acknowledgments

We are grateful to the Karonga and Lilongwe communities, participants, and traditional authorities, for their engagement in this work. This study would also not have been possible without the encouragement and support of the Malawi Ministry of Health, particularly the Karonga and Lilongwe District Health Offices.

For peer review only

For peer review only

References

1. Ogurtsova K, da Rocha Fernandes JD, Huang Y, Linnenkamp U, Guariguata L, Cho NH, et al. IDF Diabetes Atlas: Global estimates for the prevalence of diabetes for 2015 and 2040. Diabetes Res Clin Pract. 2017 Jun;128:40–50.

2. Hilawe EH, Yatsuya H, Kawaguchi L, Aoyama A. Differences by sex in the prevalence of diabetes mellitus, impaired fasting glycaemia and impaired glucose tolerance in sub-Saharan Africa: a systematic review and meta-analysis. Bull World Health Organ. 2013 Sep 1;91(9):671–682D.

3. NCD Risk Factor Collaboration - Africa Working Group. Trends in obesity and diabetes across Africa from 1980 to 2014: an analysis of pooled population-based studies. Int J Epidemiol [Internet]. 2017 [cited 2017 Jul 19]; Available from: http://fdslive.oup.com/www.oup.com/pdf/production_in_progress.pdf

4. Mbanya JCN, Motala AA, Sobngwi E, Assah FK, Enoru ST. Diabetes in sub-Saharan Africa. The Lancet. 2010 Jun;375(9733):2254–66.

5. Burgess PI, Allain TJ, García-Fiñana M, Beare N a. V, Msukwa G, Harding SP. High prevalence in Malawi of sight-threatening retinopathy and visual impairment caused by diabetes: identification of population-specific targets for intervention. Diabet Med J Br Diabet Assoc. 2014 Dec;31(12):1643–50.

For peer review only

6. The International Expert Committee. International Expert Committee Report on the Role of the A1C Assay in the Diagnosis of Diabetes. Diabetes Care. 2009 Jul 1;32(7):1327–34.

7. American Diabetes Association. Diagnosis and Classification of Diabetes Mellitus. Diabetes Care. 2014 Jan 1;37(Supplement_1):S81–90.

8. World Health Organization. Use of glycated haemoglobin (HbA1c) in diagnosis of diabetes mellitus: abbreviated report of a WHO consultation [Internet]. 2011 [cited 2018 Feb 10]. Available from: http://www.who.int/diabetes/publications/report-hba1c_2011.pdf

9. Nakanga W, Crampin A, Nyirenda M. Personal View: Should haemoglobin A1C be used for diagnosis of diabetes mellitus in Malawi? Malawi Med J. 2016 Apr 6;28(1):28.

10. Son JI, Rhee SY, Woo J, Hwang JK, Chin SO, Chon S, et al. Hemoglobin A1c May Be an Inadequate Diagnostic Tool for Diabetes Mellitus in Anemic Subjects. Diabetes Metab J. 2013;37(5):343.

11. Kim C, Bullard KM, Herman WH, Beckles GL. Association Between Iron Deficiency and A1C Levels Among Adults Without Diabetes in the National Health and Nutrition Examination Survey, 1999-2006. Diabetes Care. 2010 Apr 1;33(4):780–5.

For peer review only

12. Little RR, Roberts WL. A review of variant hemoglobins interfering with hemoglobin A1c measurement. J Diabetes Sci Technol. 2009 May 1;3(3):446–51.

13. Slama L, Palella FJ, Abraham AG, Li X, Vigouroux C, Pialoux G, et al. Inaccuracy of haemoglobin A1c among HIV-infected men: effects of CD4 cell count, antiretroviral therapies and haematological parameters. J Antimicrob Chemother. 2014 Dec 1;69(12):3360–7.

14. Glesby MJ, Hoover DR, Shi Q, Danoff A, Howard A, Tien P, et al. Glycated haemoglobin in diabetic women with and without HIV infection: data from the Women's Interagency HIV Study. Antivir Ther. 2010;15(4):571–7.

15. Herman WH. Do Race and Ethnicity Impact Hemoglobin A1c Independent of Glycemia? J Diabetes Sci Technol. 2009 Jul;3(4):656–60.

16. Cavagnolli G, Pimentel AL, Freitas PAC, Gross JL, Camargo JL. Effect of ethnicity on HbA1c levels in individuals without diabetes: Systematic review and meta-analysis. PLoS One. 2017 Feb 13;12(2):e0171315.

17. Crampin AC, Kayuni N, Amberbir A, Musicha C, Koole O, Tafatatha T, et al. Hypertension and diabetes in Africa: design and implementation of a large population-based study of burden and risk factors in rural and urban Malawi. Emerg Themes Epidemiol. 2016 Dec;13(3).

For peer review only

18. Msyamboza KP, Mvula CJ, Kathyola D. Prevalence and correlates of diabetes mellitus in Malawi: population-based national NCD STEPS survey. BMC Endocr Disord. 2014 May 12;14:41.

19. Crampin AC, Dube A, Mboma S, Price A, Chihana M, Jahn A, et al. Profile: The Karonga Health and Demographic Surveillance System. Int J Epidemiol. 2012 Jun 1;41(3):676–85.

20. World Health Organization, International Diabetes Federation. Definition and diagnosis of diabetes mellitus and intermediate hyperglycemia : report of a WHO/IDF consultation [Internet]. Geneva, Switzerland: World Health Organization; 2006. Available from: http://www.who.int/diabetes/publications/diagnosis_diabetes2006/en/

21. Youden WJ. Index for rating diagnostic tests. Cancer. 1950 Jan;3(1):32–5.

22. Mayega RW, Guwatudde D, Makumbi FE, Nakwagala FN, Peterson S, Tomson G, et al. Comparison of fasting plasma glucose and haemoglobin A1c point-of-care tests in screening for diabetes and abnormal glucose regulation in a rural low income setting. Diabetes Res Clin Pract. 2014 Apr;104(1):112–20.

23. Bertran EA, Berlie HD, Taylor A, Divine G, Jaber LA. Diagnostic performance of HbA_{1c} for diabetes in Arab vs. European populations: a systematic review and meta-analysis. Diabet Med. 2017 Feb;34(2):156–66.

For peer review only

24. Yan S, Liu S, Zhao Y, Zhang W, Sun X, Li J, et al. Diagnostic accuracy of HbA_{1c} in diabetes between Eastern and Western. Eur J Clin Invest. 2013 Jul;43(7):716–26.

25. Kodama S, Horikawa C, Fujihara K, Hirasawa R, Yachi Y, Yoshizawa S, et al. Use of high-normal levels of haemoglobin A1C and fasting plasma glucose for diabetes screening and for prediction: a meta-analysis. Diabetes Metab Res Rev. 2013 Nov;29(8):680–92.

26. Hird TR, Pirie FJ, Esterhuizen TM, O'Leary B, McCarthy MI, Young EH, et al. Burden of Diabetes and First Evidence for the Utility of HbA1c for Diagnosis and Detection of Diabetes in Urban Black South Africans: The Durban Diabetes Study. Staiger H, editor. PLoS One. 2016 Aug 25;11(8):e0161966.

27. NCD Risk Factor Collaboration. Effects of diabetes definition on global surveillance of diabetes prevalence and diagnosis: a pooled analysis of 96 population-based studies with 331 288 participants. Lancet Diabetes Endocrinol. 2015 Aug;3(8):624–37.

For peer review only

Table 1. Demographic and clinical characteristics of participants in Karonga and Lilongwe, Malawi, 2013-2017.

	Total (n=3645) Median and IQR or n and %	Karonga (n=1613) Median and IQR, or n and %	Lilongwe (n=2032) Median and IQR, or n and %
Age, years	33 (25-44)	34 (26-47)	31 (24-41)
Female sex, %	63.0	58.3	67.2
Body mass index, kg/m^2	22.6 (20.5-26.0)	21.8 (20.0-23.8)	23.8 (21.2-28.0)
Normal BMI, %	61.1	81.9	57.8
Overweight BMI, %	20.4	13.8	26.1
Obese BMI, %	10.5	4.3	16.0
Systolic blood pressure, mm Hg	119 (111-129.5)	117.5 (110-127.5)	121 (112.5-131)
Diastolic blood pressure, mm Hg	72.5 (66.5-79.5)	72 (66-78)	73 (67-80.5)
Total cholesterol, mmol/l	3.8 (3.3-4.6)	3.8 (3.2-4.5)	3.9 (3.3-4.6)

For peer review only

Triglycerides, mmol/l	0.80 (0.58-1.14)	0.81 (0.58-1.12)	0.80 (0.59-1.18)
High density lipoprotein, mmol/l	1.12 (0.94-1.31)	1.09 (0.91-1.28)	1.15 (0.98-1.34)
Low density lipoprotein, mmol/l	2.53 (2.06-3.10)	2.44 (2.00-2.96)	2.62 (2.14-3.22)
Haemoglobin, g/dl	13.7 (12.6-14.9)	13.7 (12.6-15.0)	13.7 (12.6-14.9)
Normal haemoglobin, %	82.0	81.1	82.7
Anaemia, %	18.0	18.9	17.3
HIV positive, %	8.5	7.8	9.1
HbA_{1c} %	5.3 (4.9-5.7)	5.1 (4.7-5.5)	5.4 (5.1-5.8)
$HbA_{1c} \geq 6.5\%$ (48 mmol/mol), %	7.3	5.6	8.8
Fasting plasma glucose, mmol/l	4.7 (4.3-5.0)	4.7 (4.3-5.0)	4.7 (4.4-5.1)
FPG 6.1-7.0 mmol/L, %	1.8	1.7	1.9
FPG $\geq$ 7.0, %	1.7	1.3	2.1

FPG, Fasting plasma glucose; IRQ, Interquartile range

Aside counts, the figures are re-weighted by inverse probability of receiving HbA_{1c} testing

For peer review only

Table 2. Sensitivity, specificity, and positive and negative likelihood ratios for detecting type 2 diabetes at selected HbA_{1c} thresholds for participants in Karonga and Lilongwe, Malawi, 2013-2017.

HbA_{1c} threshold			Sensitivity (%)	Specificity (%)	Positive Likelihood Ratio	Negative Likelihood Ratio
%	mmol/ mol	SD				
6.0	42	+1.0	84.4	86.9	6.5	0.18
6.5*	48	+1.7	78.7	94.0	13.3	0.23
6.6†	49	+1.8	78.3	94.6	14.5	0.23
6.7	50	+2.0	76.5	96.0	19.0	0.25
7.4	57	+3.0	66.3	98.5	43.3	0.34
8.1	65	+4.0	54.8	99.5	117.4	0.45

SD, Standard Deviations from the mean HbA_{1c} score among diabetes-negative participants

* Diagnostic threshold recommended by American Diabetes Association, European Association for the Study of Diabetes, International Diabetes

For peer review only

Federation and World Health Organization.

† Threshold identified by Youden's J statistic

Figures are re-weighted by the inverse probability of receiving HbA_{1c} testing

For peer review only

Table 3. Overall and stratum-specific area under the receiver operating characteristic (AUROC) curves for detection of type 2 diabetes with HbA_{1c} among adults in Malawi, 2013-2017.

	AUROC (95% CI)	Sensitivity (%)*	Specificity (%)*	Positive Likelihood Ratio*	Negative Likelihood Ratio*
Overall	0.92 (0.90-0.94)	78.7	94.0	13.3	0.23
Site					
Karonga	0.88 (0.94-0.92)	70.2	95.4	15.2	0.31
Lilongwe	0.94 (0.92-0.96)	83.3	92.9	11.7	0.18
Sex					
Female	0.93 (0.91-0.95)	79.1	95.2	16.5	0.22
Male	0.89 (0.85-0.93)	77.6	92.1	9.9	0.24
Body mass index					
Normal	0.87 (0.83-0.92)	70.1	95.1	14.5	0.31
Overweight	0.93 (0.89-0.96)	84.5	91.4	9.9	0.17

For peer review only

Obese	0.92 (0.89-0.95)	80.4	90.6	8.6	0.22
Haemoglobin					
Normal	0.93 (0.91-0.95)	81.4	93.5	12.6	0.20
Anaemia	0.86 (0.79-0.93)	61.5	96.4	17.4	0.40

* At HbA1c ≥ 6.5%

Figures are re-weighted by the inverse probability of receiving HbA_{1c} testing

Table 4. Clinical characteristics of participants identified as having diabetes by fasting blood glucose, and for true- and false-positive participants by HbA_{1c}≥6.5% in Karonga and Lilongwe, Malawi, 2013-2017.

	Type 2 diabetes positive by FPG ≥7.0 mmol/L (IQR)	Type 2 diabetes true positive by HbA_{1c}≥6.5%* (IQR)	Type 2 diabetes false positive by HbA_{1c}≥6.5%*† (IQR)
Age, years	50 (41-60)	51 (43-61)	35 (23-51)
Female sex, %	65.6	66.3	51.0

For peer review only

Body mass index, kg/m^2	27.9 (24.2-32.5)	28.2 (24.8-32.5)	24.8 (20.9-28.3)
Systolic blood pressure, mm Hg	133.5 (121.5-153)	135 (123-156)	125 (115-136)
Diastolic blood pressure, mm Hg	81.5 (74.5-89.5)	81.5 (75.0-90.5)	74.5 (68.5-82.5)
Total cholesterol, mmol/L	4.9 (4.1-5.7)	5.0 (4.3-5.8)	4.2 (3.5-5.2)
Triglycerides, mmol/L	1.7 (1.2-2.3)	1.8 (1.2-2.5)	0.9 (0.6-1.5)
High density lipoprotein, mmol/L	1.04 (0.89-1.22)	1.03 (0.88-1.20)	1.15 (0.97-1.29)
Low density lipoprotein, mmol/L	3.38 (2.69-4.09)	3.52 (2.89-4.22)	2.82 (2.36-3.56)
Haemoglobin, g/dl	13.9 (12.9-14.9)	13.9 (12.9-15.0)	14.2 (13.1-15.1)
HbA_{1c}, %	8.4 (6.9-11.3)	9.6 (7.7-11.8)	6.9 (6.6-7.4)
Fasting plasma glucose, mmol/L	9.5 (7.7-14.2)	10.7 (8.3-15.4)	4.8 (4.5-5.1)

FPG, Fasting Plasma Glucose; IQR, Interquartile range

For peer review only

* Diagnostic threshold recommended by American Diabetes Association, European Association for the Study of Diabetes, World Health Organization, and International Diabetes Federation.

Aside counts, figures are weighted by the inverse probability of receiving HbA_{1c} testing

For peer review only

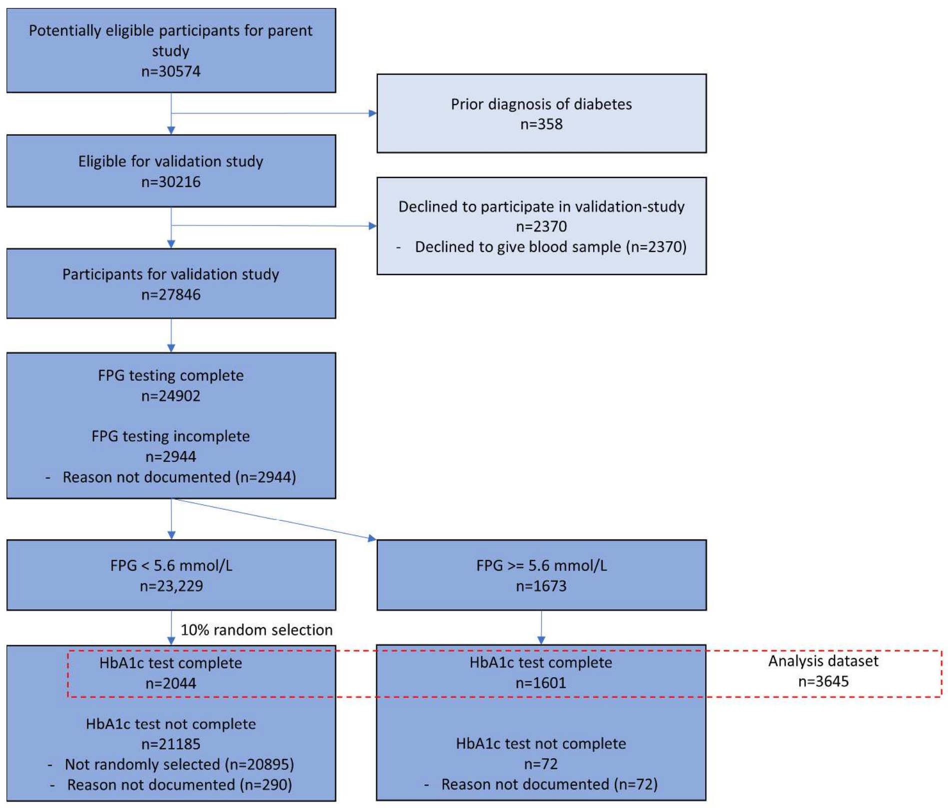

STARD diagram

250x230mm (300 x 300 DPI)

```
*******************
*** Hb1Ac AUROC ***
*******************
      use "30Aug17.dta", clear
      keep gluc_units hba1_units ///
            BMI systolic diastolic chol_units tg_units hdl ldl hgb ///
            sex site stid age
      qui compress
      sum

****************
*** NEW VARS ***
*****************************************************************
      gen diabetes_fpg=(gluc_units>=7.0)
      label variable diabetes_fpg "Newly diabetes positive (fasting plasma glucose>=7)"
      label define yesno 0 No 1 Yes
      label values diabetes_fpg yesno

      recode gluc_units (min/6.09=0)(6.10/6.99=1)(7.0/max=0), gen(prediabetes)
      label variable prediabetes "Pre-diabetes (fasting glucose of 6.1-6.9)"
      label values prediabetes yesno

      recode gluc_units (min/5.59=0)(5.6/max=1), gen(gluc56)
      label variable gluc56 "Fasting glucose >=5.6 mmol/L"
      label values gluc56 yesno

      recode hba1 (min/6.49=0)(6.50/max=1), gen(hba65)
      label variable hba65 "Diabetes screen positive (HbA1c >= 6.5%)"
      label values hba65 yesno

      recode hba1 (min/6.79=0)(6.80/max=1), gen(hba68)
      label variable hba68 "Diabetes screen pos (HbA1c >=6.8%)"
      label values hba68 yesno

      gen hbaint = round(hba1_units,1)
      label variable hbaint "HbA1c score, rounded to integer"

      gen hbadec = round(hba1_units,0.1)
```

For peer review only

```
label variable hbaint "HbA1c score, rounded to tenths"

encode sex, gen(gender)
label define gender 1 Female 2 Male, modify
label values gender gender
label variable gender "Sex"
drop sex
rename gender sex

gen tempsite = site=="L"
label define site 0 Karonga 1 Lilongwe, modify
label values tempsite site
drop site
rename tempsite site
label variable site "Research site"

gen hbatest = !mi(hba1)
gen pw =!mi(hba1)
recode pw (1=10) if gluc_units<5.6
label variable pw "Probability sampling weight for HbA1c testing"

sort stid
gen pid = _n
label variable pid "Unique identifier"
expand=pw
summarize hba1_unit if diabetes_fpg==0
gen meanhba = r(mean)
gen sdhba = r(sd)
gen zscore = (hba1_units - meanhba) / sdhba
label variable zscore "HbA1c z-score"
notes zscore: Using only HbA1c scores from diabetes-negative pts and re-weighted for the sub-sampling
duplicates drop pid, force

gen zdec = round(zscore, 0.1)
label variable zdec "Normal HbA1c z-score, rounded to 0.1"

recode BMI (min/24.9999=0)(25/29.999=1)(30/max=2), gen(bmi_3c)
label define bmi 0 Normal 1 Overweight 2 Obese
```

For peer review only

```
        label values bmi_3c bmi
        label variable bmi_3c "BMI category"

        gen anemia=0
        replace anemia=1 if sex==2 & hgb<13.5
        replace anemia=1 if sex==1 & hgb<12.0
        label values anemia yesno

        quiet compress

        tempfile full
        save `full'
*** END NEW VARS *********************************************

*** FLOW CHART ***
        use `full', clear
        count
        drop if mi(gluc_units)
        drop if mi(hba1)
        tab gluc56, sum(hba1)

**************************
*** DESCRIPTIVE CHECKS ***
**************************
        sum age sex site hba1 gluc_units diabetes_fpg pw    /* Most of these are skewed */
        logistic diabetes_fpg hba1_units [pw=pw]
        prop diabetes_fpg
        prop diabetes_fpg [pw=pw]


****************************************
*** TABLE 1: Patient characteristics ***
****************************************
        tabstat age sex BMI syst dias chol_units tg_units hdl_units ldl_units hgb hba1_units gluc_units[aw=pw],
stat(n p50 p25 p75) by(site) f(%9.2f) col(s)
        foreach var of varlist sex bmi_3c anemia hba65 prediabetes diabetes_fpg {
                prop `var' [pw=pw]
                prop `var' [pw=pw], over(site)
```

```
		}
	replace prediabetes=. if gluc_units>=7.0

*******************************
*** TABLE 2: Validity stats ***
*******************************
	* Overall dataset for HbA1c vs diabetes
	foreach x of varlist diabetes_fpg prediabetes {
		somersd `x' hba1 [pw=pw], trans(c)
		roctab `x' hba1, binomial sum
		senspec `x' hbadec [pw=pw], sensitivity(sens) specificity(spec) ntpos(tp) nfpos(fp) ntneg(tn)
nfneg(fn)
		for var sens spec tp fp tn fn: replace X = 100*X
		gen ppv = 100*tp / (tp+fp)
		gen npv = 100*tn / (tn+fn)
		gen lrpos = sens / (100-spec)
		gen lrneg = (100-sens) / spec
		gen jtemp = sens + spec - 100
		egen youdenj = max(jtemp)
		replace youdenj=. if youdenj!=jtemp
		table zdec, c(mean sens mean spec mean youdenj mean lrpos mean lrneg) format(%9.2f)
		drop sens spec tp fp tn fn ppv npv lrpos lrneg jtemp youdenj
	}

***************
*** TABLE 3 ***
***************
	* Stratum-specific HbA1c vs diabetes
	foreach var of varlist site sex bmi_3c anemia {
		levelsof `var', local(levels)
		foreach l of local levels {
			tab `var' if `var'==`l'
			somersd diabetes_fpg hba1 [pw=pw] if `var'==`l', trans(c)
			senspec diabetes_fpg hbadec [pw=pw] if `var'==`l', sensitivity(sens) specificity(spec)
			for var sens spec: replace X = 100*X
			gen lrpos = sens / (100-spec)
			gen lrneg = (100-sens) / spec
```

For peer review only

```
                    table zdec if zdec>1.66 & zdec<1.71 & `var'==`l', c(mean sens mean spec mean lrpos mean
lrneg) format(%9.2f)
                    drop sens spec lrpos lrneg
                    }
             }

*******************************
*** Table 4: Diabetes chars ***
*******************************
* Present clinical charteristics of diabetes+ by fasting glucose criteria
      tabstat age sex BMI syst dias chol_units tg_units hdl_units ldl_units hgb hba1_units gluc_units  if
diabetes_fpg [aw=pw], stat(p50 p25 p75) f(%9.2f) col(s)
      prop sex [pw=pw] if diabetes_fpg

* Present clinical characteristics among HbA1c+, by true status
      tab hba65 diabetes_fpg
      tabstat age sex BMI syst dias chol_units tg_units hdl_units ldl_units hgb hba1_units gluc_units  if hba65
[aw=pw], by(diabetes_fpg) stat(p50 p25 p75) f(%9.2f) col(s)
      prop sex if hba65 [pw=pw], over(diabetes_fpg)
```

For peer review only

Section & Topic	No	Item	Reported on page #
**TITLE OR ABSTRACT**			
	**1**	Identification as a study of diagnostic accuracy using at least one measure of accuracy (such as sensitivity, specificity, predictive values, or AUC)	1
**ABSTRACT**			
	**2**	Structured summary of study design, methods, results, and conclusions (for specific guidance, see STARD for Abstracts)	3
**INTRODUCTION**			
	**3**	Scientific and clinical background, including the intended use and clinical role of the index test	5
	**4**	Study objectives and hypotheses	6
**METHODS**			
Study design	**5**	Whether data collection was planned before the index test and reference standard were performed (prospective study) or after (retrospective study)	6
Participants	**6**	Eligibility criteria	6
	**7**	On what basis potentially eligible participants were identified (such as symptoms, results from previous tests, inclusion in registry)	6
	**8**	Where and when potentially eligible participants were identified (setting, location and dates)	6
	**9**	Whether participants formed a consecutive, random or convenience series	6
Test methods	**10a**	Index test, in sufficient detail to allow replication	7
	**10b**	Reference standard, in sufficient detail to allow replication	7
	**11**	Rationale for choosing the reference standard (if alternatives exist)	15
	**12a**	Definition of and rationale for test positivity cut-offs or result categories of the index test, distinguishing pre-specified from exploratory	8
	**12b**	Definition of and rationale for test positivity cut-offs or result categories of the reference standard, distinguishing pre-specified from exploratory	7
	**13a**	Whether clinical information and reference standard results were available to the performers/readers of the index test	7
	**13b**	Whether clinical information and index test results were available to the assessors of the reference standard	7
Analysis	**14**	Methods for estimating or comparing measures of diagnostic accuracy	8
	**15**	How indeterminate index test or reference standard results were handled	NA
	**16**	How missing data on the index test and reference standard were handled	NA
	**17**	Any analyses of variability in diagnostic accuracy, distinguishing pre-specified from exploratory	8
	**18**	Intended sample size and how it was determined	6
**RESULTS**			
Participants	**19**	Flow of participants, using a diagram	Figure 1
	**20**	Baseline demographic and clinical characteristics of participants	11
	**21a**	Distribution of severity of disease in those with the target condition	NA
	**21b**	Distribution of alternative diagnoses in those without the target condition	NA
	**22**	Time interval and any clinical interventions between index test and reference standard	6
Test results	**23**	Cross tabulation of the index test results (or their distribution) by the results of the reference standard	10-11
	**24**	Estimates of diagnostic accuracy and their precision (such as 95% confidence intervals)	11
	**25**	Any adverse events from performing the index test or the reference standard	11
**DISCUSSION**			
	**26**	Study limitations, including sources of potential bias, statistical uncertainty, and generalisability	15
	**27**	Implications for practice, including the intended use and clinical role of the index test	12-13
**OTHER INFORMATION**			
	**28**	Registration number and name of registry	NA
	**29**	Where the full study protocol can be accessed	NA
	**30**	Sources of funding and other support; role of funders	16

For peer review only
